# Role of heterotrimeric Gα proteins in maize development and enhancement of agronomic traits

**DOI:** 10.1371/journal.pgen.1007374

**Published:** 2018-04-30

**Authors:** Qingyu Wu, Michael Regan, Hiro Furukawa, David Jackson

**Affiliations:** Cold Spring Harbor Laboratory, Cold Spring Harbor, NY, United States of America; University of Minnesota, UNITED STATES

## Abstract

Plant shoot systems derive from the shoot apical meristems (SAMs), pools of stems cells that are regulated by a feedback between the WUSCHEL (WUS) homeobox protein and CLAVATA (CLV) peptides and receptors. The maize heterotrimeric G protein α subunit COMPACT PLANT2 (CT2) functions with CLV receptors to regulate meristem development. In addition to the sole canonical Gα CT2, maize also contains three eXtra Large GTP-binding proteins (XLGs), which have a domain with homology to Gα as well as additional domains. By either forcing CT2 to be constitutively active, or by depleting XLGs using CRISPR-Cas9, here we show that both CT2 and XLGs play important roles in maize meristem regulation, and their manipulation improved agronomic traits. For example, we show that expression of a constitutively active CT2 resulted in higher spikelet density and kernel row number, larger ear inflorescence meristems (IMs) and more upright leaves, all beneficial traits selected during maize improvement. Our findings suggest that both the canonical Gα, CT2 and the non-canonical XLGs play important roles in maize meristem regulation and further demonstrate that weak alleles of plant stem cell regulatory genes have the capacity to improve agronomic traits.

## Author summary

Maize is one of the most important cereal crops worldwide. Optimizing its yields requires fine tuning of development. Therefore, it is critical to understand the developmental signaling mechanisms to provide basic knowledge to maximize productivity. The heterotrimeric G proteins transmit signals from cell surface receptor and have been shown to regulate many biological processes, including shoot development. Here we study the role of G protein α subunits in maize development by either making the only canonical Gα constitutively active or mutating all other non-canonical Gα subunits (XLGs). We demonstrate that CT2 and XLGs have both redundant and specialized functions in regulating shoot development. Importantly, we show that a constitutively active *G*α functioned as a weak allele, which introduced multiple desirable agronomic traits, such as improved kernel row number and reduced leaf angle.

## Introduction

The plant shoot system is derived from the SAMs, pools of stems cells that have the ability of self-renewal, while initiating new leaves and axillary meristems [1]. The CLV-WUS negative feedback loop has been identified as the key pathway to regulate SAM proliferation and differentiation in *Arabidopsis*, and is widely conserved in other species [2]. This pathway relies on the communication between a battery of receptors, peptides and transcription factors. WUS, a homeodomain transcription factor expressed in the organizing center, promotes stem cell fate [2], while CLV3, a small peptide ligand that is secreted from stem cells at the tip of the SAM, is perceived by leucine-rich repeat (LRR) receptor kinases, such as CLV1, and receptor-like protein CLV2, resulting in the repression of WUS transcription [3–5]. The CLV pathway is conserved in crops, for example maize CLV1 and CLV2 receptor orthologs THICK TASSEL DWARF1 (TD1) and FASCIATED EAR2 (FEA2) function in meristem regulation, and both *td1* and *fea2* mutants show enlarged meristems, or fasciated, phenotypes [6, 7]. However, the signaling players and mechanisms downstream of the CLV receptors are poorly understood.

A common class of proteins that signal directly downstream of cell surface receptors in mammalian systems is the heterotrimeric G proteins. These proteins, consisting Gα, Gβ, and Gγ subunits, are also key regulators in the transduction of extracellular signals in plants [8]. The classical model established in animals suggests that in the inactive state, the GDP-bound Gα associates with the Gβγ dimer. Ligand activation of an associated 7-transmembrane domain (7-TM) G-protein-coupled receptor (GPCR) induces the exchange of GDP for GTP on Gα, promoting dissociation of Gα from the receptor and Gβγ dimer. The activated Gα and Gβγ subunits then interact with downstream effectors to transduce signaling [9]. Emerging evidence suggests that instead of interacting with 7-TM GPCRs as in animals, the plant G proteins interact with single-TM receptors to regulate plant development and disease resistance [10–13]. Recent genetic screens in maize and *Arabidopsis* identified roles for heterotrimeric G protein α and β subunits in meristem regulation, by interacting with CLV related receptors [10, 12]. In maize, the Gα subunit COMPACT PLANT2 (CT2) interacts *in vivo* with the LRR receptor-like protein FEA2, to control shoot and inflorescence meristem development. *ct2* mutants have enlarged SAMs, fasciated ears with enlarged ear inflorescence meristems and more rows of kernels [10]. In contrast, in *Arabidopsis* the Gβ subunit, AGB1, interacts with another CLV-related receptor RECEPTOR-LIKE PROTEIN KINASE2 (RPK2), to transmit the stem cell restricting signal, and *agb1* mutants develop bigger SAMs [12].

In addition to interacting with a different class of receptors, the regulatory mechanism of Gα function in plants appears to be fundamentally different from that in animals, since plant Gα subunits spontaneously exchange GDP for GTP *in vitro*, without requiring GPCR activation [14, 15]. This novel mechanism of regulation involves a non-canonical Regulator of G-protein Signaling (RGS) protein in *Arabidopsis*, which contains a 7-TM domain coupled to an RGS domain [16], and promotes conversion of Gα-GTP back to Gα-GDP [16]. However, RGS homologs are missing from many grass species, including maize [15, 17–19]; therefore, the mechanism of plant G protein regulation, particularly the transition between the active and inactive states, remain largely unknown in these species. Expression of constitutively active Gα subunits that have lost GTPase activity, disrupting the balance between active and inactive Gα, results in distinct phenotypes, supporting the idea that Gα activity needs to be carefully controlled [16, 20, 21]. However, the implication of Gα constitutive activity on meristem development has not been addressed.

Plants also differ from animals in containing only a relatively small number of heterotrimeric G protein genes. Most plants have only one canonical Gα [15], however they also encode non-canonical Gα subunits, extra-large GTP binding proteins (XLGs), which contain a Gα domain at the C-terminus [22–28]. *Arabidopsis* has 3 XLGs, and they function either redundantly or independently, depending on the biological process [22–28]. *Arabidopsis xlg1/2/3* triple mutants do not have an obvious shoot meristem phenotype, however knocking out the 3 *XLGs* in a Gα (*gpa1*) background leads to a significant increase in shoot meristem size [25], suggesting they function redundantly with the canonical Gα in meristem regulation; however, the importance of G protein signaling in diverse plant species remains obscure. Taking advantage of the strong developmental phenotypes of maize Gα mutant *ct2*, here we explore the roles of G proteins in maize development by either making Gα constitutively active or mutating all maize XLGs using multiplex CRISPR-Cas9. We demonstrate that CT2 and XLGs have both redundant and specialized functions in regulating meristem development, and importantly, manipulation of maize Gα subunits introduced desirable agronomic traits.

## Results

### Constitutively active CT2/Gα behaves like a weak allele

**Fig 1:**
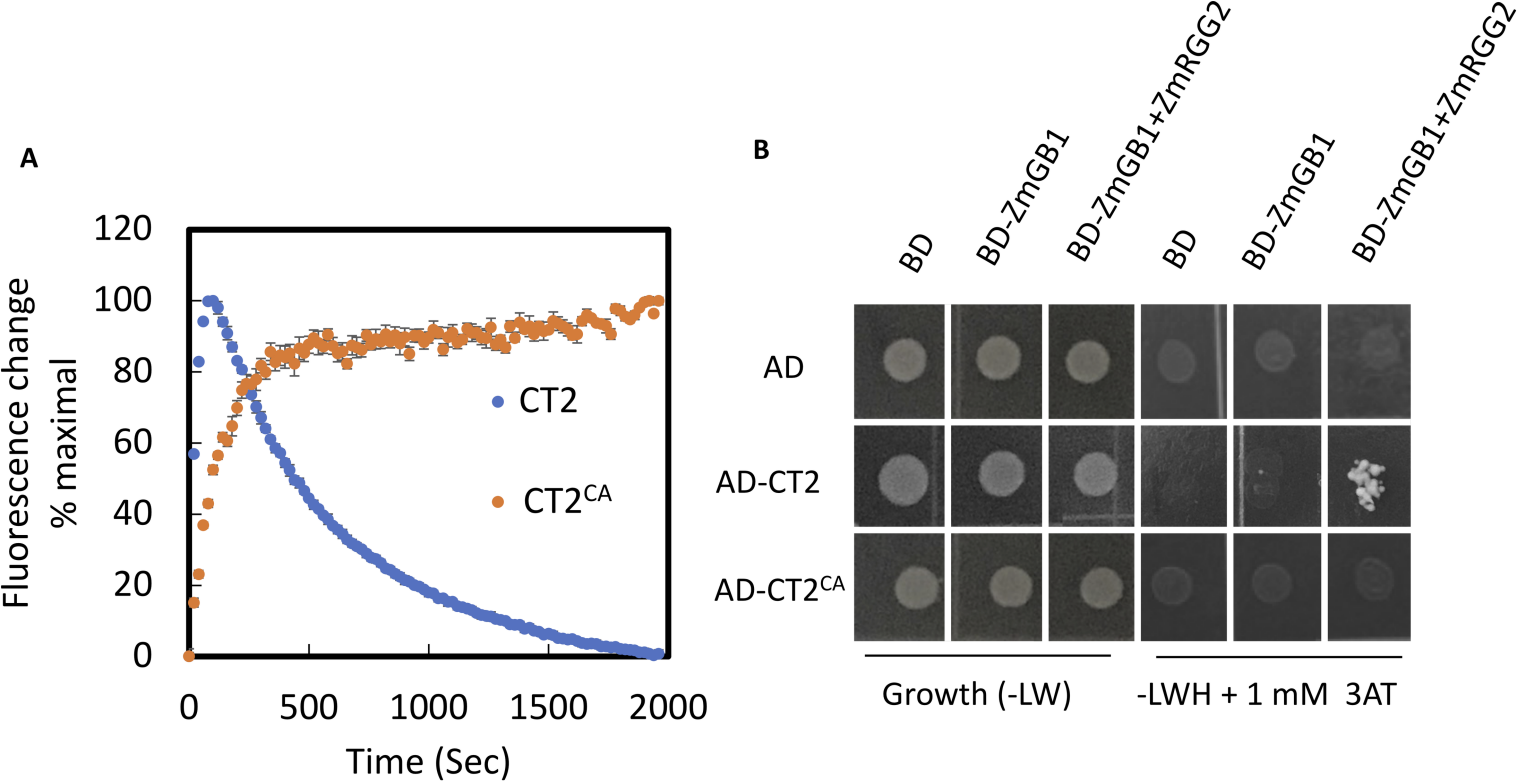
Q223L point mutation resulted in a constitutively active CT2 (CT2^CA^). **(A)** BODIPY-GTP assay for detecting the GTP-binding and GTPase activity of His-CT2 and His-CT2^CA^ proteins. Data are shown as means of four replicates and error bars represent S. D. **(B)** CT2^CA^ did not interact with a Gβγ dimer in a Y3H assay. Yeast growth on synthetic complete -Trp -Leu (-LW) medium confirmed transformation and cell viability. Interactions were assayed on SC -Trp -Leu -His (-LWH) medium supplemented with 1 mM 3-AT.

Our previous study showed that the maize heterotrimeric G protein α subunit CT2 plays an important role in shoot meristem regulation, by associating with a maize CLV receptor FEA2 [10]. However, the underlying signaling mechanism remains obscure, and the implication of Gα activity on meristem development has not been addressed. We took the opportunity of the strong maize phenotype to investigate the effect of forcing Gα to be constitutively active *in vivo*. We hypothesize that the GTPase activity and the GDP-GTP exchange cycle are required for full Gα function in transmitting the CLV signaling to regulate maize meristem development, and thus mutants that are defective in GTPase activity may act as a weak allele of *ct2*. Exchange of a single amino acid in mammalian, *Arabidopsis*, or rice Gα proteins is sufficient to block GTP hydrolysis, resulting in a constitutively active (GTPase-dead) form [16, 20, 29]. On this basis, we introduced an analogous point mutation, Q223L, in *CT2*, to generate a constitutively active protein, which we named CT2^CONSTITUTIVELY ACTIVE^ (CT2^CA^). To ask if the Q223L mutation abolished GTPase activity, we performed *in vitro* GTP-binding and GTPase activity assays using fluorescent BODIPY-GTP, where an increase in fluorescence over time corresponds to GTP binding, and a subsequent decrease corresponds to GTP hydrolysis [30]. We first established that CT2 works as an authentic Gα protein, by testing GTP/GDP binding and hydrolysis specificity. CT2 rapidly bound then slowly hydrolyzed fluorescent GTP, with similar kinetics to other vascular plant Gα proteins (**Fig 1A and S1A Fig**) [15, 30], and the activity was efficiently competed by non-labeled GTP or GDP but not by ATP or ADP (**S1C Fig**). As expected, the CT2^CA^ protein had similar GTP-binding, but lacked GTPase activity (**Fig 1A and S1D Fig**). We further asked if CT2^CA^ interacted with Gβγ in a yeast-3-hybrid (Y3H) system. In contrast to CT2, we found that CT2^CA^ did not interact with the Gβγ dimer, despite being expressed at a similar level as CT2 in the yeast cells (**Fig 1B and S1B Fig**). In summary, the Q223L point mutation abolished the GTPase activity of CT2, maintaining it in a constitutively active state that could no longer form a heterotrimeric complex with Gβγ.

**Fig 2:**
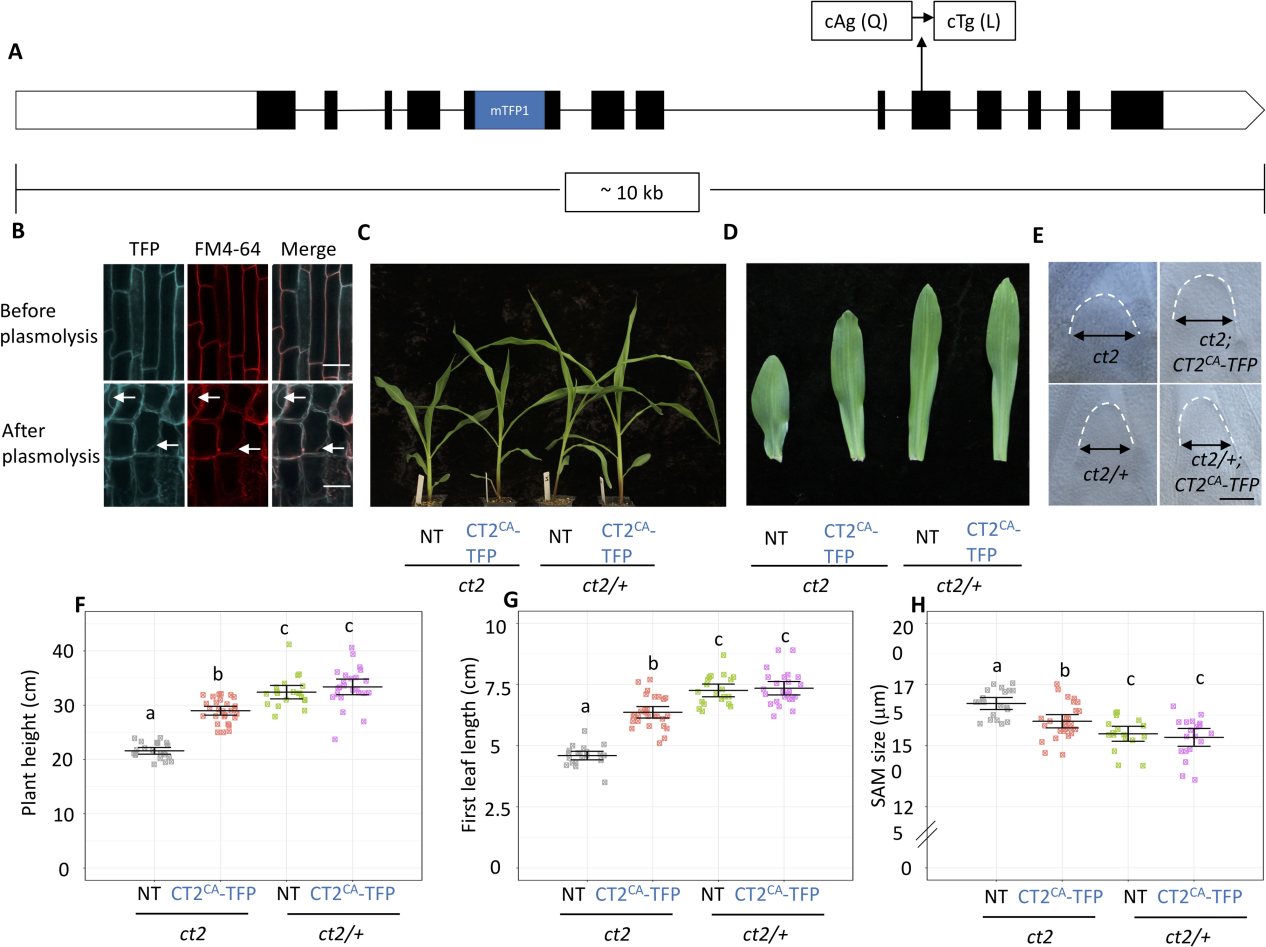
Expression of *CT2*^*CA*^*-mTFP1* partially complemented the vegetative growth of *ct2* mutants. **(A)** The *CT2*^*CA*^*-mTFP1* construct in a native context of the *CT2* genomic region. **(B)** CT2^CA^-mTFP1 was co-localized with FM4-64 on the plasma membrane, scale bar = 50 μm. Expression of *CT2*^*CA*^*-mTFP1* partially complemented the *ct2* dwarf phenotype **(C and F)**, the leaf length phenotype **(D and G)**, and the enlarged SAM size phenotype **(E and H)**, scale bar = 100 μm. NT, non-transgenic control. The raw values are shown in **(F-H)**, the horizontal black lines indicate the means, and the error bars represent 95% confidence intervals; for (**F and G**) n = 21, 28, 20, and 24, respectively; for (**H**) n = 20, 27, 18, and 20, respectively. Data were analyzed using ANOVA followed by the LSD test. The groups containing the same letter are not significantly different at the *p*-value of 0.05.

To test if constitutive activation of CT2 impacted maize development, we introduced the Q223L point mutation into a native *CT2* expression construct that also carried an in-frame fusion of *mTFP1* at an internal position that maintains full protein function [10] (**Fig 2A**). After transformation into maize, CT2^CA^-mTFP1 was correctly localized in a thin line at the cell periphery that co-localized with a plasma membrane (PM) counterstain, FM4-64 (**Fig 2B**), and we confirmed this co-localization following plasmolysis (**Fig 2B**). We next backcrossed 6 independent transgenic events of *CT2*^*CA*^*-mTFP1* into *ct2* mutants in a B73 background. Our previous work established that a native *CT2-YFP* expression construct fully complements *ct2* mutant phenotypes, and we found that both *CT2*^*CA*^*-mTFP1* and *CT2-YFP* were expressed at a similar level as the endogenous *CT2* [10] (**S2A, S2B and S2D Fig**). We first asked if *CT2*^*CA*^*-mTFP1* was able to complement the vegetative growth defects of *ct2* mutants, by measuring plant height and the first leaf length. *CT2*^*CA*^*-mTFP1; ct2* plants were significantly taller than *ct2* mutants, with longer leaves; however, they were significantly smaller than their normal, *ct2* heterozygous siblings with or without the *CT2*^*CA*^*-mTFP1* transgene, indicating that *CT2*^*CA*^*-mTFP1* only partially rescued the vegetative growth defects of *ct2* mutants (**Fig 2C, 2D, 2F and 2G**, similar results obtained with 6 independent transgenic events, **S2C Fig**). We also asked if *CT2*^*CA*^*-mTFP1* could complement the enlarged meristem phenotypes of *ct2* mutants. We again found partial complementation, indicating that CT2^CA^ was only partially functional in meristem regulation (**Fig 2E and 2H**). Since CT2 is involved in the CLV-WUS pathway by interacting with FEA2 [10], we tested if CT2^CA^ can still interact with FEA2 in an *N*. *benthamiana* transient expression system. The result showed that FEA2-Myc was pulled down by both CT2-YFP and CT2^CA^-YFP in the co-IP experiment (**S3 Fig**). Similarly, studies in human and insect cells showed that in some cases G protein subunits and receptors remain associated following receptor activation [31–33]. Further studies will be needed to elucidate the underlying mechanisms. In addition, to ask how CT2^CA^ affected downstream signaling, we measured *ZmWUS1* expression in inflorescence transition stage meristems by qRT-PCR. However, we found that *ZmWUS1* expression was not significantly changed in *ct2* mutants compared to wild type, nor in our constitutively active *CT2*^*CA*^*-mTFP1* lines (**S4 Fig**), similar to other studies involving subtle changes in CLV pathway genes [34, 35] and reflecting the complex non-linear regulation of the CLV-WUS negative feedback loop. Collectively, our results suggest that *ct2*^*ca*^ functioned as a weak allele of *ct2*, and that normal GTPase activity and the GDP-GTP exchange cycle is required for full Gα function in maize.

### ZmXLGs function in maize development

**Fig 3:**
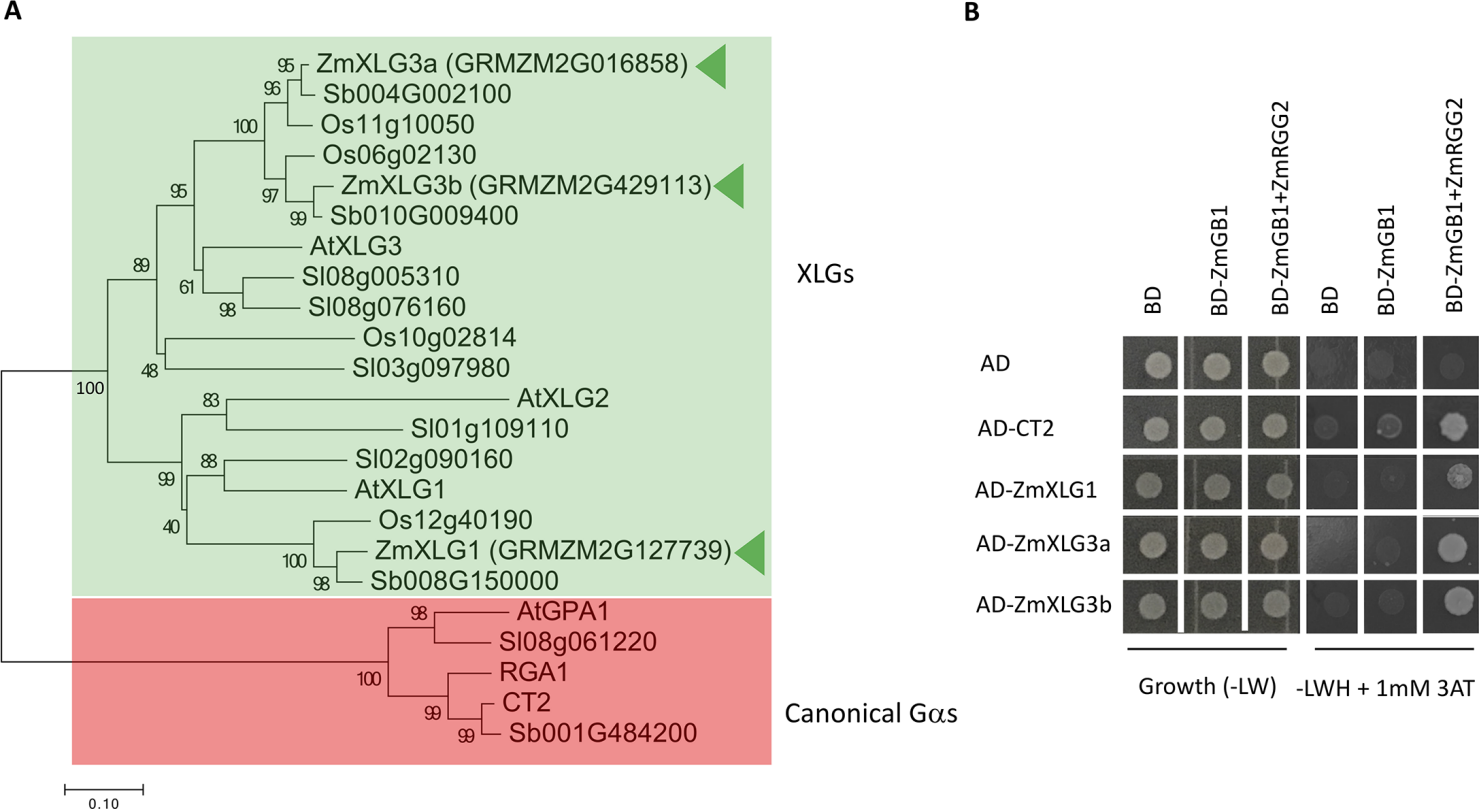
ZmXLGs function within a heterotrimeric G protein complex. **(A)** A neighbor-joining phylogenetic tree for canonical Gα’s and three XLGs in maize. Numbers at the branch points represent bootstrap values obtained by 100 iterations. Branches of XLGs and canonical Gαs were highlighted by green and red colors, respectively. **(B)** Maize XLGs interacted with a Gβγ dimer in a Y3H assay. Yeast growth on synthetic complete (SC) -Trp -Leu (-LW) medium confirmed transformation and cell viability. Interactions were assayed on SC -Trp -Leu -His (-LWH) medium supplemented with 1 mM 3-AT.

We next asked if CT2/Gα function in maize might be compensated by XLGs. We used phylogenetic analysis (**Fig 3A**) to compare the maize XLGs to *Arabidopsis*, and based on this named them ZmXLG1 (most similar to AtXLG1) and ZmXLG3a and b (most similar to AtXLG3). We first asked if the three ZmXLGs might function in a heterotrimeric G protein complex, by testing their interaction with a Gβγ dimer in Y3H system. All three were indeed able to interact, similar to CT2, suggesting that they function in maize heterotrimeric G protein complexes (**Fig 3B**).

**Fig 4:**
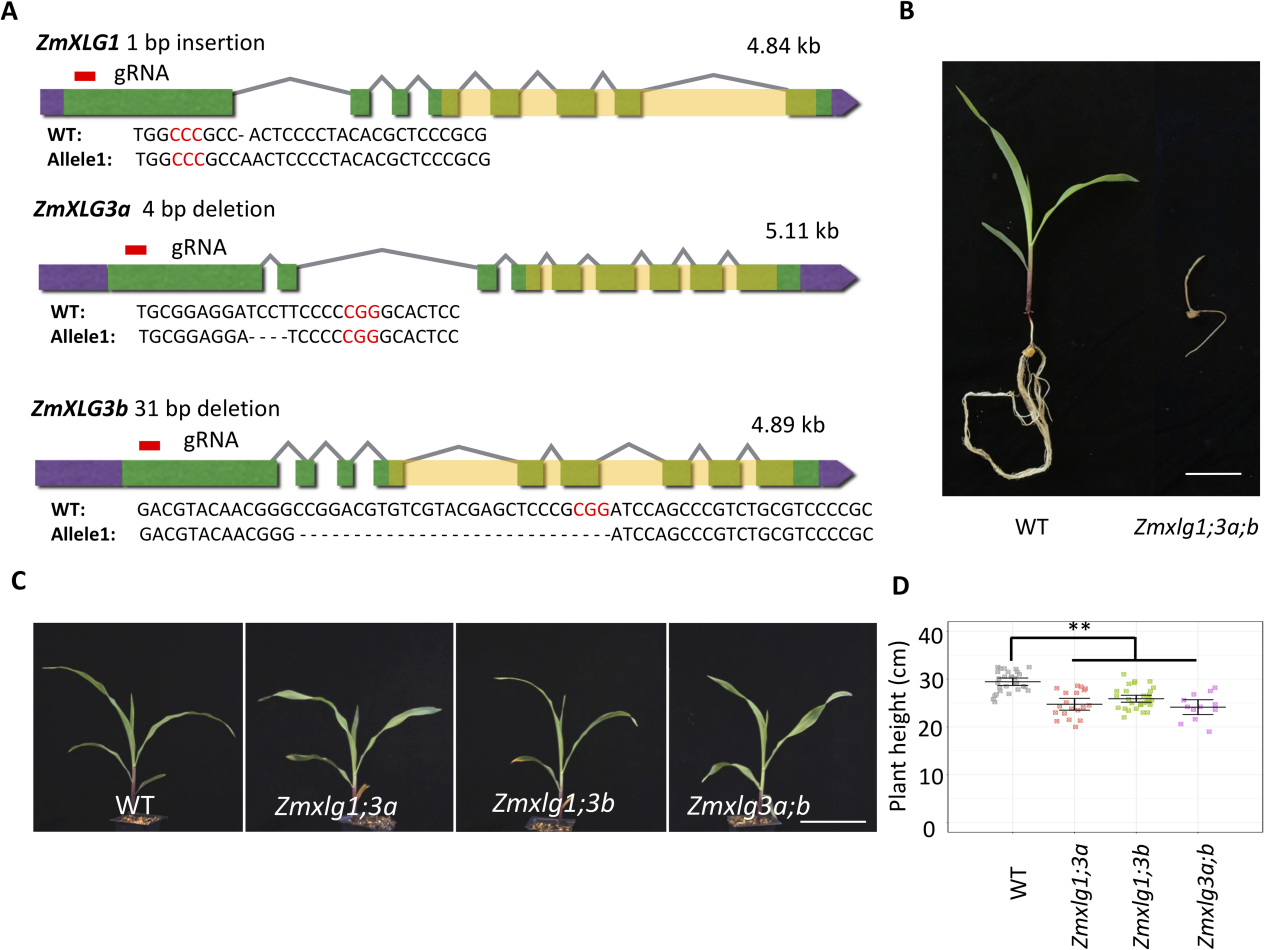
Knocking out *ZmXLGs* led to developmental phenotypes. **(A)** Generating lesions for *ZmXLGs* using CRISPR-Cas9. Red lines indicate the position of guide RNAs. 5’ and 3’-UTRs indicated in purple, exons indicated green, introns indicated by lines, and Gα domains are shaded. **(B)** *Zmxlg1;3a;b* triple mutants were lethal at the seedling stage. Scale bar = 5 cm. **(C and D)** Knocking out *ZmXLGs* reduced plant height, scale bar = 10 cm. Data were analyzed using ANOVA followed by the LSD test. ** means *p*-value < 0.01. The raw values are shown in **(D)**, the horizontal black lines indicate the means, and the error bars represent 95% confidence intervals; n = 29, 19, 33, and 12, respectively.

To study the functions of ZmXLGs in maize development, we knocked out all three genes using a tandem guide RNA (gRNA) CRISPR-Cas9 construct. In one transgenic event, we recovered putative null alleles of all 3 genes, a 1-bp insertion allele for *ZmXLG1*, a 4-bp deletion allele for *ZmXLG3a*, and a 31-bp deletion allele for *ZmXLG3b*, each within the N terminal half of the protein coding region and before the Gα domain (**Fig 4A**). Inbreeding these plants produced offspring homozygous for all 3 loci, at the expected ratio. All *Zmxlg* triple mutant plants showed a striking developmental arrest phenotype, as they were lethal at the seedling stage (**Fig 4B**), much more severe than in *Arabidopsis*, where the triple mutants are smaller with reduced fertility, but can still complete the life cycle [27, 36]. To gain a deeper understanding into the lethal phenotype, we assayed for cell death using trypan blue staining. As shown in **S5A Fig**, the triple mutants had strong staining, suggesting they were undergoing cell death. We also measured the expression of two immune marker genes, *PATHOGENESIS-RELATED PROTEIN 1* (*PR1)* and *PR5*, and found both were significantly up-regulated in the triple mutants, indicating that the lethality may be due to over-activation of immune system (**S5B Fig**). Rice *G*β mutants also display cell death and lethality [37, 38], indicating that the lethal phenotype of G protein mutants is not unique to maize. The reason for these differences between monocot and dicot G protein mutants remains elusive, but may be related to their dual role in immune signaling [13, 24, 39, 40]. Although the *Zmxlg* triple mutant plants stopped growing soon after germination, we could measure their shoot meristems, and found that they were normal in size and structure (**S6A Fig**).

**Fig 5:**
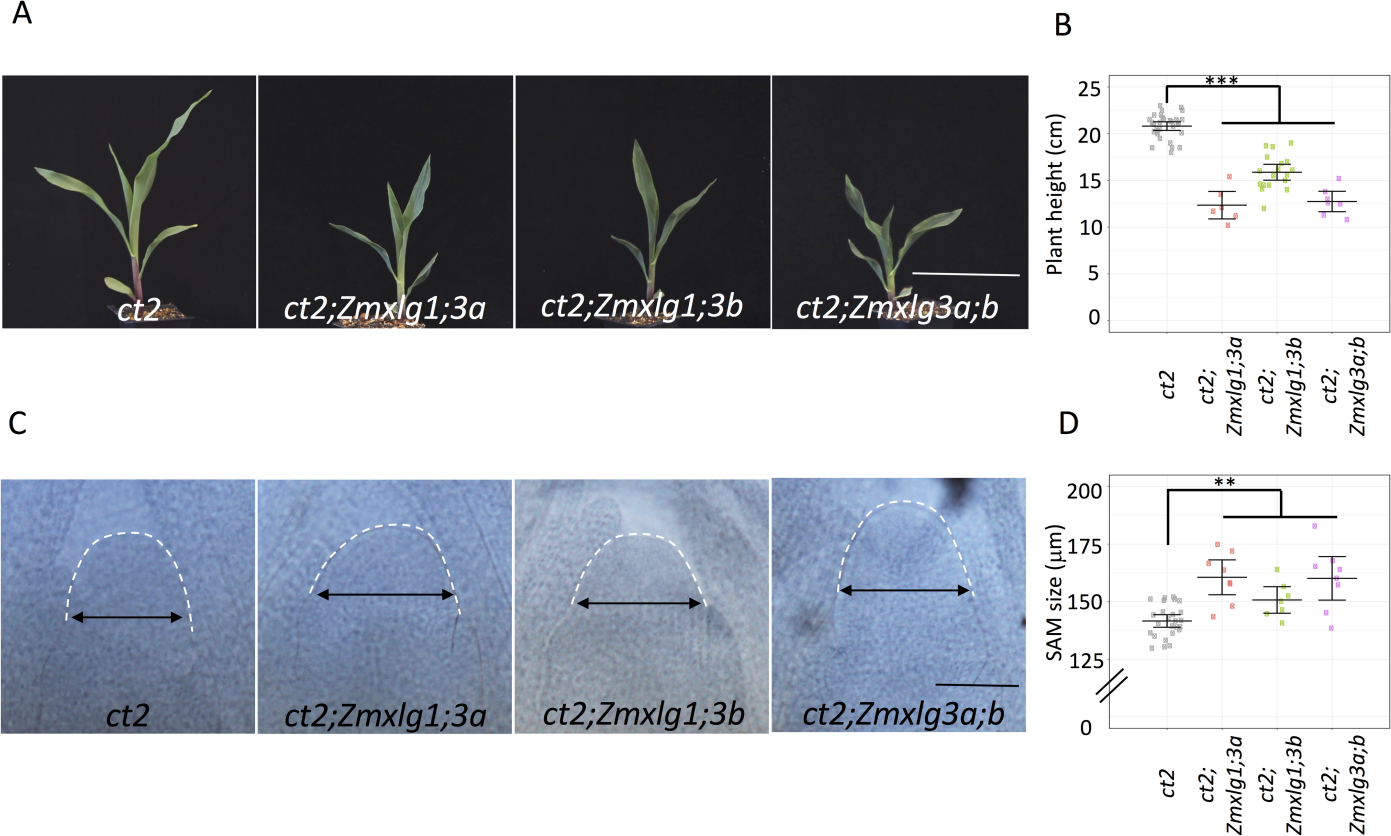
Knocking out *ZmXLGs* enhanced *ct2* phenotypes. Knocking out *ZmXLGs* in a *ct2* mutant background enhanced the dwarf phenotype **(A and B)** and increased SAM size **(C and D)**. Scale bars represent 10 cm **(A)** and 100 μm **(C**), respectively. Data were analyzed using ANOVA followed by the LSD test. ** means *p*-value < 0.01, *** means *p*-value < 0.001. The raw values are shown in **(B and D)**, the horizontal black lines indicate the means, and the error bars represent 95% confidence intervals; for **(B)** n = 31, 6, 18, and 7, respectively; for **(D)** n = 25, 8, 7, and 8, respectively.

As the *Zmxlg1;3a;b* triple mutants were lethal, we next analyzed the developmental phenotype of single or double mutants. Knocking out each single *ZmXLG* did not alter development; whereas knocking out any two *ZmXLGs* led to a modest but significant reduction in plant height, but did not affect SAM size (**Fig 4C and 4D and S7 Fig**), indicating that loss of any two *ZmXLGs* can be partially compensated by other XLGs or by CT2/Gα. Next, we asked if ZmXLGs function redundantly with the canonical maize Gα, CT2, by crossing the *Zmxlg* mutants into a *ct2* mutant background. As expected, *ct2* mutants were significantly shorter than wild-type siblings [10], and we found that mutation of any two *ZmXLGs* dramatically enhanced their dwarf phenotype (**Fig 5A and 5B**). In addition, mutation of any pair of *ZmXLGs* significantly increased SAM size in a *ct2* mutant background (**Fig 5C and 5D**), indicating that ZmXLGs are partially redundant with CT2 in SAM regulation. In contrast, although both *CT2* and *ZmXLGs* are expressed in the maize inflorescence, *ZmXLG* knockouts did not enhance the *ct2* inflorescence fasciation phenotype (**S6B, S6C and S8 Figs**), suggesting that *CT2* is the major Gα functioning in inflorescence meristem development. In summary, our results showed that XLGs are partially redundant with CT2 at some stages of development, but that all 3 XLGs redundantly function in early maize development, where they are essential for survival past the germination stage, and cannot be compensated by *CT2*.

### Weak Gα phenotypes provide desirable agronomic traits

**Fig 6:**
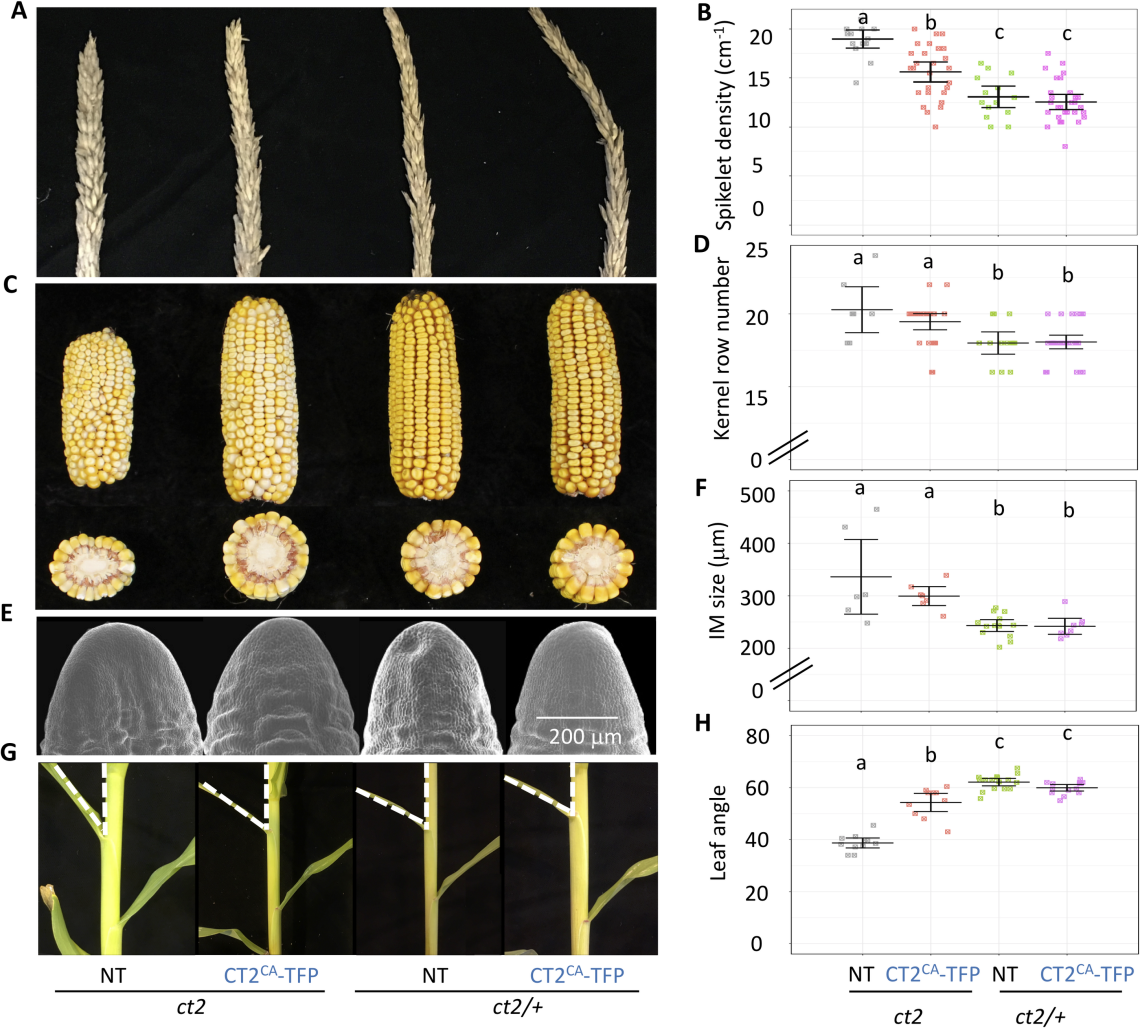
Expression of *CT2*^*CA*^*-mTFP1* enhanced agronomic traits. Expression of *CT2*^*CA*^*-mTFP1* in a *ct2* mutant background increased spikelet density (**A and B**), KRN **(C and D),** and ear inflorescence meristem (IM) size **(E and F)**. Expression of *CT2*^*CA*^*-mTFP1* in a *ct2* mutant background also significantly reduced the leaf angle **(G and H)**. The raw values are shown in **(B, D, F, H**), the horizontal black lines indicate the means, and the error bars represent 95% confidence intervals; for **(B)** n = 15, 27, 14, and 29, respectively; for **(D)** n = 7, 26, 13, and 28, respectively; for **(F)** n = 6, 7, 14, and 8, respectively; for **(H)** n = 11, 10, 16, and 14, respectively. Data were analyzed using ANOVA followed by the LSD test. The groups containing the same letter were not significantly different at the *p*-value of 0.05. NT, non-transgenic control.

Our previous results indicate that weak alleles of meristem regulatory genes, such as *fea2* or *fea3* can improve agronomic traits, such as increasing kernel row number (KRN), without the negative yield impacts associated with strong fasciation phenotypes [41, 42]. The results described above suggest that different *Zmxlg* mutant combinations reduce maize height, which is an important trait selected during breeding of many cereal crops [43, 44]. We also found that *ct2*^*ca*^ functions as a weak allele of *CT2*, and therefore asked if its expression might affect agronomic traits. First, we measured tassel spikelet density, a trait associated with increased meristem size [10, 42], of *CT2*^*CA*^*-mTFP1*-expressing plants in a *ct2* homozygous or heterozygous background. *ct2* plants expressing *CT2*^*CA*^*-mTFP1* had a significantly higher spikelet density compared with normal, *ct2* heterozygous siblings with or without the *CT2*^*CA*^*-mTFP1* transgene (**Fig 6A and 6B**). In addition, these plants did not develop stunted, fasciated ears as in *ct2* mutants, but made ears of normal length with increased KRN compared with normal, *ct2* heterozygous siblings with or without the *CT2*^*CA*^*-mTFP1* transgene (**Fig 6C and 6D**). Since our previous results suggest that there is a positive correlation between the ear inflorescence meristem size and kernel row number [41], we next checked if this is also true for *ct2* plants expressing *CT2*^*CA*^*-mTFP1*. Consistently, we found that they had significantly larger ear IMs compared with normal, *ct2* heterozygous siblings with or without the *CT2*^*CA*^*-mTFP1* transgene (**Fig 6E and 6F**), but were not fasciated.

Leaf angle is another important agronomic trait, because more upright leaves reduce shading and improve photosynthetic efficiency in modern high plant density production systems[45]. *ct2* mutants have more erect leaves, however also have negative pleiotropic traits such as extreme dwarfing and very wide leaves [10, 46, 47]. Interestingly, we found that plants expressing constitutively active CT2 also had a more erect leaf angle compared with normal, *ct2* heterozygous siblings with or without the *CT2*^*CA*^*-mTFP1* transgene, without obvious negative pleiotropic phenotypes (**Fig 6G and 6H**). In summary, we found that *ct2* plants expressing a constitutively active CT2/Gα develop phenotypes consistent with a weak allele of *ct2*. These finding suggest that the GTPase activity and the GDP-GTP exchange cycle is required for full CT2 function *in vivo*, but that expression of a constitutively-active version of *CT2* can act as a partially functional (weak) allele that brings desirable agronomic traits.

## Discussion

Heterotrimeric G protein signaling in mammals and yeast transmits a plethora of developmental and physiological signals from GPCRs to downstream effectors [48, 49]. Mammals contain many Gα homologs, therefore the full significance of knocking out all Gα signaling has not been addressed. Plants contain a much smaller number, usually a single canonical Gα and ~3 related XLGs [9, 15, 22, 50]. XLGs are evolved from the canonical Gα, and share some redundant functions [25]. In some extreme examples such as moss *Physcomitrella patens*, the canonical Gα even has been lost during evolution, and its function has been completely replaced by the sole XLG [51]. XLGs have also gained independent functions during evolution, for example, *Arabidopsis* XLG2, but not the canonical Gα, interacts with the FLS2 receptor and mediates flg22-induced immune responses [13]. In *Arabidopsis*, knockouts of all 3 XLGs have no obvious effect on shoot meristem development, and the additional knockout of the canonical Gα leads to only modest effects on development, including a change in leaf shape and slightly larger shoot meristem [25]. These results suggest that the canonical Gα and XLGs work redundantly to regulate shoot development, and heterotrimeric G protein signaling plays a relatively modest role in plant development. In this report, we found that the maize XLGs work both redundantly and independently with the canonical Gα, CT2. *Zmxlg* mutations enhanced *ct2* null phenotypes in plant height and meristem size during vegetative development, suggesting ZmXLGs function redundantly with CT2 in SAM regulation. However, knocking out all the 3 *XLGs* in maize leads to a striking early seedling growth arrest and lethality, independent of the presence of *CT2*, suggesting ZmXLGs are essential in maize early growth and development. In addition, knocking out *ZmXLGs* did not enhance ear fasciation, suggesting CT2 is the sole Gα functioning in inflorescence meristem development. Collectively, our results suggest that the maize XLGs and CT2 have overlapping functions at certain stage of development, however, both have evolved specialized functions. While we do not know the signaling pathways of the maize XLGs, it is likely that they interact with receptors involved in plant growth and development, analogous to the interaction between Gα and a CLV receptor [10].

The classic heterotrimeric G protein model established in the mammalian system suggests that Gα is usually in the inactive GDP-bound state, and is activated to switch to the active GTP-bound state by ligand binding to a 7-TM GPCR [9]. However, the plant G proteins, including those from grasses, are spontaneously active *in vitro*, and it is still under debate if plants have canonical 7-TM GPCRs [15]. Instead, several single TM receptors, such as CLV and innate immune receptors have been found to interact with G proteins [10–13]. Recent studies in *Arabidopsis* suggest that turning off plant Gα signaling is also an important step for its signal transduction [15, 52], indicating that the balance between the active and inactive Gα pool is important to fully exert its function. We found that native expression of *CT2*^*CA*^*-mTFP1* in maize partially rescued *ct2* mutant phenotypes. Sometimes partial transgene complementation of a mutant is due to improper transgene expression. However, our native *CT2-YFP* expression construct fully complemented *ct2* mutant phenotypes, and *CT2*^*CA*^*-mTFP1* was expressed at the same level as *CT2-YFP* and endogenous *CT2* (**S2D Fig**), so we conclude that the partial complementation is indeed caused by the loss of GTPase activity. In yeast, a constitutively active Gα also similarly only partially complemented the growth defects of Gα null mutants [53], suggesting that the requirement for GTPase activity is universal. In addition to GTPase activity, GTP binding is also important for the function of Gα. For example, the T475N mutant of *Arabidopsis* XLG2, which lacks GTP binding activity, is not able to interact with a downstream effector RELATED TO VERNALIZATION1 (RTV1) [54]. Together, all of these studies suggest that Gα has to bind GTP and to cycle between the active and inactive state to fully exert its function. One explanation for the importance of the cycling is that the Gα controls meristem development through coordinating with the Gβγ dimer pool. Presumably, in both *ct2* mutants and *CT2*^*CA*^ background, more free Gβγ dimers are released. *Arabidopsis* Gβ regulates the meristem development via interacting with a CLV-like receptor RPK2 [12], while the maize Gα, CT2 interacts with another CLV receptor-like protein, FEA2 [10]. It is possible that Gα and Gβ function independently by coordinating with different receptors and downstream effectors at the cell surface, whereas signaling converges at some point. Although their downstream effectors remain largely unknown in plants, Gβ forms a complex with mitogen-activated protein kinases (MAPKs) [40], which may function in the CLV pathway [55]. Therefore, fine-tuning of the active and inactive states of G protein as well as the Gα and Gβγ pools may be important to maintain meristem development, and our study illustrates the complexity of G protein signaling in meristem regulation. Importantly, *ct2*^*ca*^ functioned as a weak allele and introduced desirable agronomic phenotypes, similar to many weak alleles that underlie QTLs for crop traits [41, 42, 56]. Optimization of traits such as spikelet density, kernel row number, and leaf angle has been key to improvements in maize and other crops, both in improving yield per plant and planting density. Targeting specific regulators such as Gα by using CRISPR to generate weak alleles could enhance multiple yield related phenotypes to meet the food demands of the increasing global population.

## Materials and methods

### Y3H assay

Yeast codon-optimized ORFs of *CT2* (GRMZM2G064732), *CT2*^*CA*^, *ZmXLG1* (GRMZM2G127739), *ZmXLG3a* (GRMZM2G016858), and *ZmXLG3b* (GRMZM2G429113) were cloned between the *EcoRI* and *XhoI* restriction sites of MCS1 of pGADT7 (Clontech). *ZmGB1* (GRMZM2G045314) was cloned between the *EcoRI* and *BamHI* restriction sites of MCS1. *ZmRGG2 (GRMZM6G935329)* was cloned between the *NotI* and *BglII* restriction sites of MCS2 of pBRIDGE (Clontech), respectively. The primer sequences are shown in the supplementary information. The yeast assay was performed in the AH109 yeast strain (Clontech). The double transformants were selected on SC -Trp -Leu (-LW) plates. The interaction was tested on the SC -Trp -Leu -His (-LWH) medium supplemented with 1 mM 3-Amino-1,2,4-triazole (3-AT) to suppress histidine synthesis. The HA-tag was detected using the monoclonal anti-HA antibody produced in mouse (Sigma, clone HA-7).

### Knocking out *ZmXLGs* using CRISPR-Cas9

The guide RNAs were designed using the CRISPR-P website (http://cbi.hzau.edu.cn/crispr/) [57]. The multi-gRNA array was synthesized and cloned into pMGC1005 vector by the LR recombination reaction (Invitrogen) (**S1 File**) [58]. The construct was introduced into EHA101 and transformed into HiII background using *Agrobacterium*-mediated transformation by Iowa State University Plant Transformation Facility. The genomic regions spanning the gRNA target sites were amplified by PCR and sequenced. The T0 plants containing lesions in all three *XLG* genes were backcrossed with *ct2* in the B73 background and self-crossed.

### Generating *CT2*^*CA*^*-mTFP1*-expressing transgenic lines

*CT2*^*CA*^*-mTFP1* was constructed by amplification of genomic fragments and fusing with the *mTFP1* gene in-frame at an internal position using the MultiSite Gateway Pro system (Invitrogen), as described [10]. All fragments were amplified using KOD Xtreme hot start polymerase (Millipore Sigma) and the Q223L point mutation was generated using PCR-based mutagenesis. The ORF of *mTFP1* was inserted between the two amino-terminal α helices, αA and αB of CT2, as described [10]. All the entry clones were assembled in the pTF101 Gateway compatible binary vectors and introduced into the EHA101 *Agrobacterium* strain. The construct was transformed into HiII background using *Agrobacterium*-mediated transformation by the Iowa State University Plant Transformation Facility. The T0 plants were backcrossed twice with *ct2* mutants in the B73 background. For genotyping, a 1.5 kb fragment of the *CT2* gene was amplified and digested with *AccI*, as a single SNP causes a loss of the 5’ *AccI* site in the *ct2*-Ref allele. The transgene was amplified using one primer against the *mTFP1* sequence and the other primer against the *ct2* sequence. Primers are listed in the **S1 Table**.

### Plant growth conditions

For the SAM, ear IM, plant height, and leaf angle measurements, the plants were grown in the greenhouse with the light cycle 16/8 h light/dark and the temperature was maintained between (26–28°C). For the spikelet density and KRN measurement, the plants were grown at the Uplands Farm Agricultural Station at Cold Spring Harbor, New York between June to October.

### SAM and ear IM measurements

For SAM measurements, maize seedlings were grown in the greenhouse for 15 days and then dissected and fixed in FAA (10% formalin, 5% acetic acid, and 45% ethanol). The fixed tissues were subsequently dehydrated with 70, 85, 95 and 100% ethanol for 30 min each and then immersed in an ethanol-methyl salicylate solution (1:1) for an additional 60 min. The tissue was then cleared in 100% methyl salicylate for 2 hours. The SAMs were imaged with a Leica DMRB microscope with a Leica MicroPublisher 5.9 RTV digital camera system. For IM measurements, ear primordia 2 mm in length were dissected. The pictures were taken using a Hitachi S-3500N scanning electron microscope or a Nikon SMZ1500 dissection microscope equipped with a camera. The SAMs and ear IMs were measured using Image J.

### qRT-PCR

The 4-week old shoot apices were harvested for measuring *ZmWUS1* (GRMZM2G047448) expression, and 1-wk old seedlings were used to measure *CT2*, *CT2^CA^-mTFP1*, and *CT2-YFP* as well as *PR1* (GRMZM2G465226) and *PR5* (GRMZM2G402631) expression. qRT-PCR was performed on a CFX96 Real-Time system (Bio-Rad). Total mRNA was extracted using the Direct-zol RNA extraction kit (Zymo Research). The cDNA was synthesized using the iScript Reverse Transcription Supermix (Bio-Rad) according to the manufacturer’s manual. The relative expression level of the targeted genes was normalized using *ZmUBIQUITIN*. Primers are listed in **S1 Table.**

### Trypan blue staining

The trypan blue staining was performed using 1-wk old wild-type and *Zmxlg* triple mutants, as described with slight modifications [59]. Briefly, the whole shoot was immersed in lactophenol containing 2.5 mg/mL trypan blue, and heat in a boiling water for 1 min. Then allowing the samples site at room temperature for overnight. The tissue was cleared in chloral hydrate solution (25 g of chloral hydrate in 10 ml of H_2_O) for 24 hours at room temperature.

### Protein expression, extraction, and co-immunoprecipitation assays

The ORF of YFP was inserted between the two amino-terminal α helices, αA and αB of CT2 or CT2^CA^, as described [10]. The entry clones containing 2x35S promoter, CT2 or CT2^CA^-YFP, and Nos terminator were assembled in the *pTF101* Gateway compatible binary vectors. The ORF of *FEA2* was fused with the *6xMyc* tag sequence and cloned into the *pEARLEY301* vector [60]. All the binary vectors were introduced into the GV3101 *Agrobacterium* and infiltrated into 4-week-old *N*. *benthamiana* leaves with a P19 vector to suppress posttranscriptional silencing [61]. The protein extraction and membrane fraction enrichment were performed as described [10] with some modifications. Briefly, the leaves were harvested 3-day post infiltration and ground in liquid nitrogen to a fine powder then suspended in twice the volume of protein extraction buffer containing 150 mM NaCl, 50 mM Tris-HCl pH 7.5, 5% glycerol, and cOmplete, mini, EDTA-free protease inhibitor (Roche) and rotated in a cold room for 15 min. Then centrifuge at 4,000g for 10 min at 4°C, followed by filtration through Miracloth (Millipore Sigma), resulting a total protein extraction. The extract was then centrifuged at 100,000g for 1 h at 4°C to pellet the microsomal membrane fraction. The resulting pellet was re-suspended in 2 ml extraction buffer supplemented with 1% Triton X-100 with a glass homogenizer. Then the lysates were cleared by centrifugation at 100,000g for 1 h at 4°C to remove non-solubilized material. For co-immunoprecipitation experiments, solubilized microsomal membrane fractions were incubated with 30 μl magnetic beads coupled to monoclonal mouse anti-GFP antibody (μMACs, Milteny Biotec, 130-094-3252) for 30 min at 4°C. Flow-through columns were equilibrated using 250 μl membrane solubilization buffer before lysates were added. The MicroBead-bound target proteins were magnetically separated, and washed one time with 250 μl membrane solubilization buffer and three times with wash buffer 1 containing 150 mM NaCl, 50 mM Tris pH7.5, 0.1% SDS and 0.05% IGEPAL-CA-630 followed by one time with wash buffer 2 containing 20 mM Tris, pH 7.5, supplied by the company. Bound target proteins were eluted with 70 μl 1xSDS loading buffer. Following standard SDS-PAGE electrophoresis and blot transfer, FEA2-Myc protein was detected using an anti-Myc antibody generated from mouse (Millipore Sigma, 05–724) and a secondary HRP-coupled anti-mouse antibody (GE Healthcare Life Sciences, NA931). CT2 or CT2^CA^-YFP proteins were detected using an HRP-conjugated anti-GFP antibody (Miltenyi Biotech, 130-091-833).

### Phylogenetic analysis

BLAST search against the protein databases of *Arabidopsis*, maize, rice, and tomato using *Arabidopsis* GPA1 and maize CT2 was conducted in Phytozome (www.phytozome.com). The sequences were aligned with Clustal X [62] and the phylogenetic tree was constructed using the neighbor-joining model of MEGA7 [63]. One hundred bootstrap iterations were performed.

### Protein purification and BODIPY-GTP assay

The coding sequence of *CT2* was cloned into pPROEX-His vector between restriction sites *EcoRI* and *XhoI*. *CT2*^*CA*^ was generated using PCR-based mutagenesis (Primers are shown in the **S1 Table**). Both constructs were transformed into Rosetta DE3 *E*.*Coli* cells for protein expression, as described by Urano et al. with modification [15]. The transformed cells were grown to an OD_600_ of 0.6 prior to induction by 0.5 mM 1-thio-β-D-galactopyranoside (IPTG) in LB medium for 18 hrs at 16°C. Cells were harvested by centrifugation and resuspended in 150 mM NaCl, 50 mM Tris, 10 mM imidazole, 5 mM β-mercaptoethanol (β-ME), 1 mM MgCl_2_, 10 μM GDP, and 10% glycerol, adjusted to a final pH of 7.5, and a cOmplete, mini, EDTA-free protease inhibitor tablet (Roche) was added. Cells were lysed by passage three times through a cell disruptor (Avestin) at greater than 15,000 psi, and the lysate was centrifuged at 29,000 g for 30 min to produce a clarified lysate. This lysate was loaded onto a cobalt-charged NTA resin column (GE Life Sciences), and washed with 500 mM NaCl, 50 mM Tris, 20 mM imidazole, 5 mM β-ME, 1 mM MgCl_2_, 10 μM GDP, and 5% glycerol, pH 7.5. Bound His-tagged protein was eluted with the same buffer including 300 mM imidazole and 10 mM MgCl_2_ prior to concentration and loading onto a Superdex-200 size-exclusion column (GE Life Sciences) equilibrated with 100 mM NaCl, 50 mM Tris, 10 mM MgCl_2_, 5 mM β-ME, 10 μM GDP, and 5% glycerol (final pH 7.5). Peak fractions were pooled and dialyzed to an appropriate buffer for later experiments.

The BODIPY-GTP assay was performed as described previously with slight modification [64]. Assays were performed at 25°C in a 200 μl reaction volume in the assay buffer (20 mM Tris-HCl, pH 8.0 and 10 mM MgCl_2_) with 25 μM purified protein and 50 nM BODIPY-GTP. For competition with non-labeled nucleotides, 25 μM of GTP, GDP, ATP or ADP was added to the assay buffer before starting the reaction. The fluorescence (excitation 485 nm, emission 528 nm) was recorded every 10 s for up to 40 min using a BioTek Synergy H4 fluorescence microplate reader.

### Microscopy

For imaging of *CT2*^*CA*^*-mTFP1*, roots were counterstained with 1 mg/ml FM4-64 solution in water for 1min and washed with water. Images were taken with a Zeiss LSM 710 microscope, using 458 nm excitation and 488–515 nm emission for detection of mTFP1 and 514 nm excitation and 585–750 nm emission for detection of FM4-64. For plasmolysis, the tissues were treated with 20% sucrose for 30 min.

### Statistical analysis

The significant differences between multiple groups were analyzed using ANOVA followed by the LSD test with Bonferroni correction in the R statistical programming language (www.R-project.org). All experiments were repeated at least twice and similar results were obtained. The result from one repetition is presented.

## Supporting information

S1 Fig Specificity of GTP binding and hydrolysis by CT2 and CT2^CA^ proteins. **(A)** Purified recombinant His-CT2 and His-CT2^CA^ proteins from *E*. *coli*. **(B)** HA-AD-CT2 and HA-AD-CT2^CA^ proteins were expressed at similar levels in yeast, by western blot. BODIPY-GTP assay for detecting the GTP-binding and GTPase activity of His-CT2 **(C)** and His-CT2^CA^ proteins **(D)**. GTP and GDP compete efficiently for fluorescent GTP binding, but ATP or ADP does not. CT2 rapidly bound then slowly hydrolyzed fluorescent GTP. The CT2^CA^ protein had similar GTP-binding, but lacked GTPase activity. Data are means of four replicates and error bars represent S.D.
(TIF)

S2 Fig Expression of *CT2*^*CA*^*-mTFP1* partially complemented the hight of *ct2* mutants. Expression of *CT2-YFP* but not of *CT2*^*CA*^*-mTFP1* fully complemented the height of *ct2* mutants (A and B). Consistent results were obtained for multiple different *CT2*^*CA*^-expressing events in a *ct2* mutant background, compared with *ct2* homozygous or heterozygous plants **(C)**. **(D)** *CT2*^*CA*^*-mTFP1*, *CT2-YFP*, and endogenous *CT2* were expressed at a similar level. Expression levels were measured by qRT-PCR, relative to *ZmUBIQUITIN*. Data are shown as means; error bars represent S.D.; data were analyzed using ANOVA followed by the Fisher’s LSD test. For **(A-C)** n = 5–10; the groups containing the same letter are not significantly different at the *p*-value of 0.05. NT, non-transgenic control. For (D) n = 3 biological replicates; each replicate contains a pool of 4 plants.
(TIF)

S3 Fig FEA2 interacts with both CT2 and CT2^CA^. FEA2-Myc was pulled down by both CT2-YFP and CT2^CA^-YFP in co-IP experiments using the membrane fractions following co-expression in *N*. *benthamiana* leaves.
(TIF)

S4 Fig *ZmWUS1* expression was not affected by CT2 constitutive activity. Data are shown as means; error bars represent S.D.; n = 3 biological replicates. The tissues were collected from 4-wk old maize shoot apices. Each replicate contains pooled samples from 4–6 plants.
(TIF)

S5 Fig The *Zmxlg* triple mutants showed cell death phenotype, and up-regulation of immune response marker genes. **(A)** Trypan blue staining of fully expanded wild-type (WT) and *Zmxlg1;3a;3b* triple mutant leaf blade showed increased staining in the triple mutants, scale bar = 3 mm. Note, the mutant leaf is smaller because of the early growth arrest. **(B)** *PR1* and *PR5* expression is massively up-regulated in the *Zmxlg* triple mutants. Values were normalized to the expression of *ZmUBIQUITIN*. Error bars represent S.D.; n = 3 biological replicates; *p* < 0.01 in a *Student’s t-test*.
(TIF)

S6 Fig The effects of ZmXLGs on SAM and IM development. **(A)** *Zmxlg123* triple mutants displayed normal shoot apical meristems. WT, wild-type. Scale bar = 100 μm. Data are shown as means; error bars represent S.D.; n.s. indicates not significantly different (*p*-value>0.05) in a student’s t-test. Knocking out *ZmXLGs* in a *ct2* mutant background did not enhance the fasciation phenotype of either tassel **(B)** or ear **(C)** primordia. Scale bar = 500 μm.
(TIF)

S7 Fig Knocking out *ZmXLGs* did not affect the SAM size. Scale bar = 100 μm. Data were analyzed using ANOVA followed by the Fisher’s LSD test. n.s. indicates not significantly different (*p*-value>0.05). Data are shown as means; error bars represent S.D.; n = 9–23.
(TIF)

S8 Fig Expression of *CT2* and *ZmXLGs* in the maize inflorescence other tissues. **(A)** Expression of *CT2* and *ZmXLGs* in the maize inflorescence. The data were mined from www.maizeinflorescence.org and Reference 1 in S1 File Eveland et al., 2014. **(B)** Expression of *CT2* and *ZmXLGs* in the different tissues at different developmental stages. The data were mined from www.maizegdb.com and Reference 2 in S1 File Stelpflug et al., 2016.
(TIF)

S1 Table List of the primer sequences. (DOCX)

S1 File Sequence of the ZmXLG multiple-gRNA array and references for supporting information. (DOCX)
