# Supplementary figures and images for: Role of heterotrimeric Gα proteins in maize development and enhancement of agronomic traits
Source: PLoS Genet. 2018 Apr 30;14(4):e1007374. doi: 10.1371/journal.pgen.1007374 (PMC5945058; doi:10.1371/journal.pgen.1007374)

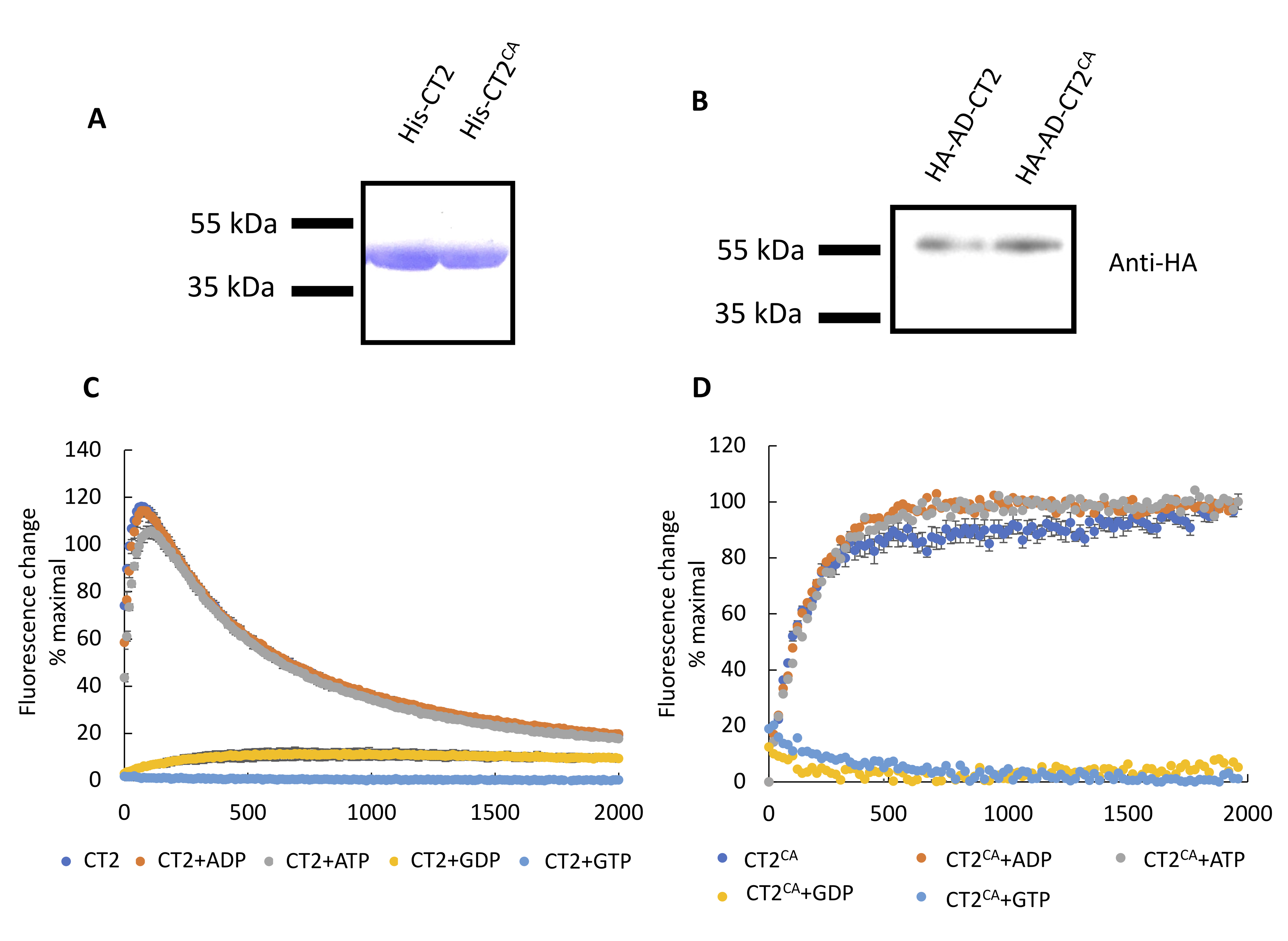

Supplement: S1 Fig — (A) Purified recombinant His-CT2 and His-CT2CA proteins from E. coli. (B) HA-AD-CT2 and HA-AD-CT2CA proteins were expressed at similar levels in yeast, by western blot. BODIPY-GTP assay for detecting the GTP-binding and GTPase activity of His-CT2 (C) and His-CT2CA proteins (D). GTP and GDP compete efficiently for fluorescent GTP binding, but ATP or ADP does not. CT2 rapidly bound then slowly hydrolyzed fluorescent GTP. The CT2CA protein had similar GTP-binding, but lacked GTPase activity. Data are means of four replicates and error bars represent S.D. (TIF) [file pgen.1007374.s001.tif]

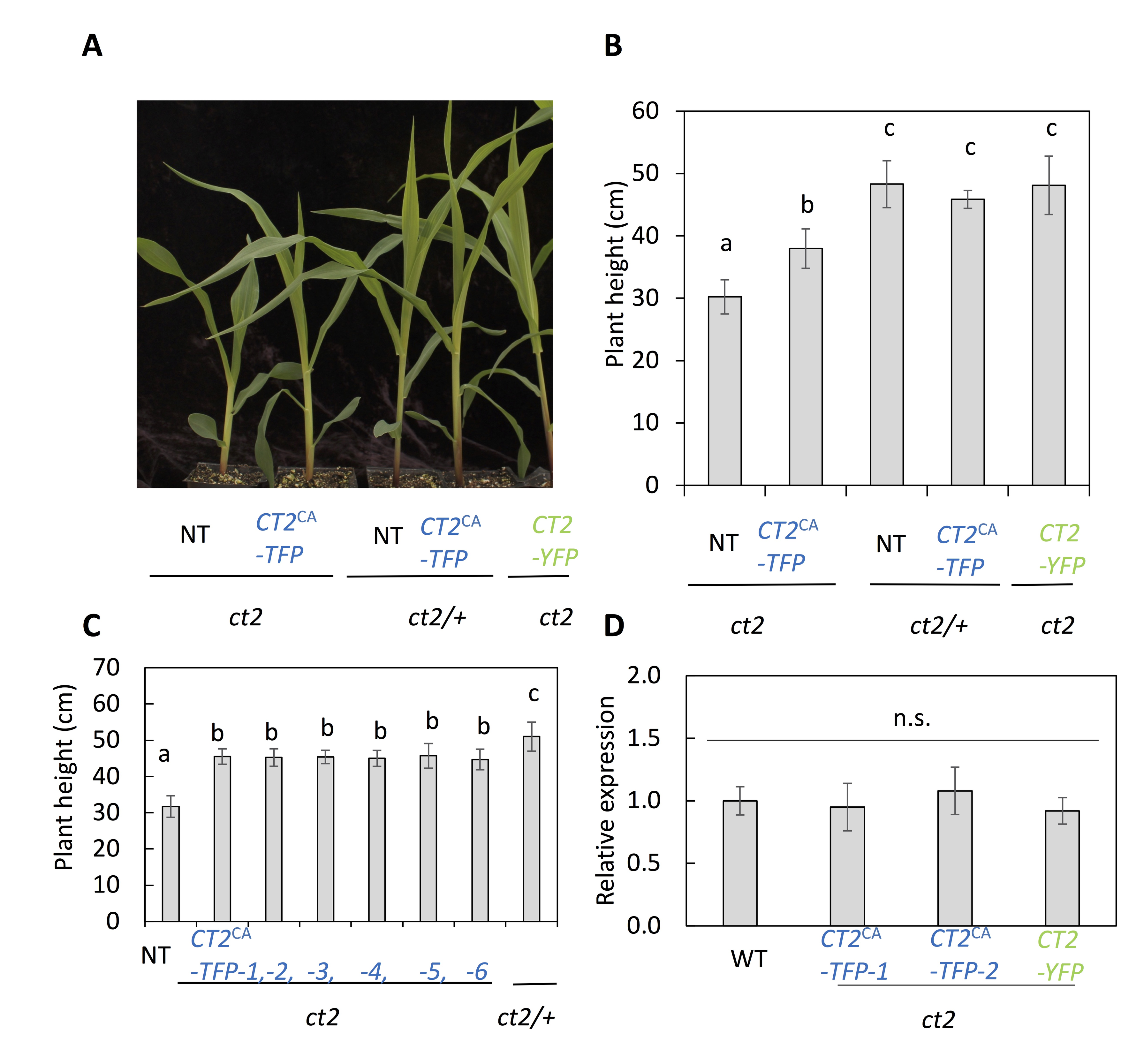

Supplement: S2 Fig — Expression of CT2-YFP but not of CT2CA-mTFP1 fully complemented the height of ct2 mutants (A and B). Consistent results were obtained for multiple different CT2CA-expressing events in a ct2 mutant background, compared with ct2 homozygous or heterozygous plants (C). (D) CT2CA-mTFP1, CT2-YFP, and endogenous CT2 were expressed at a similar level. Expression levels were measured by qRT-PCR, relative to ZmUBIQUITIN. Data are shown as means; error bars represent S.D.; data were analyzed using ANOVA followed by the Fisher’s LSD test. For (A-C) n = 5–10; the groups containing the same letter are not significantly different at the p-value of 0.05. NT, non-transgenic control. For (D) n = 3 biological replicates; each replicate contains a pool of 4 plants. (TIF) [file pgen.1007374.s002.tif]

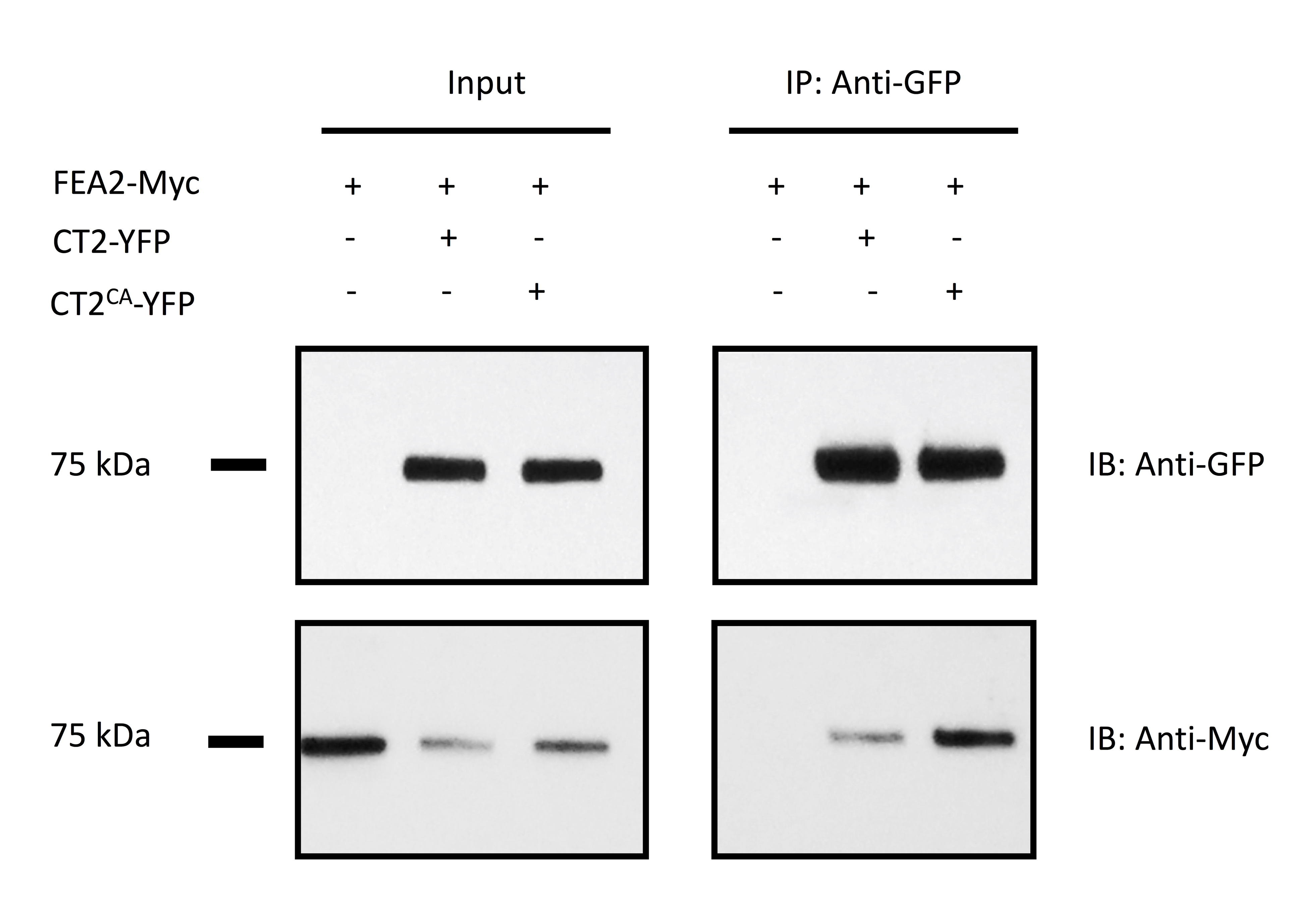

Supplement: S3 Fig — FEA2-Myc was pulled down by both CT2-YFP and CT2CA-YFP in co-IP experiments using the membrane fractions following co-expression in N. benthamiana leaves. (TIF) [file pgen.1007374.s003.tif]

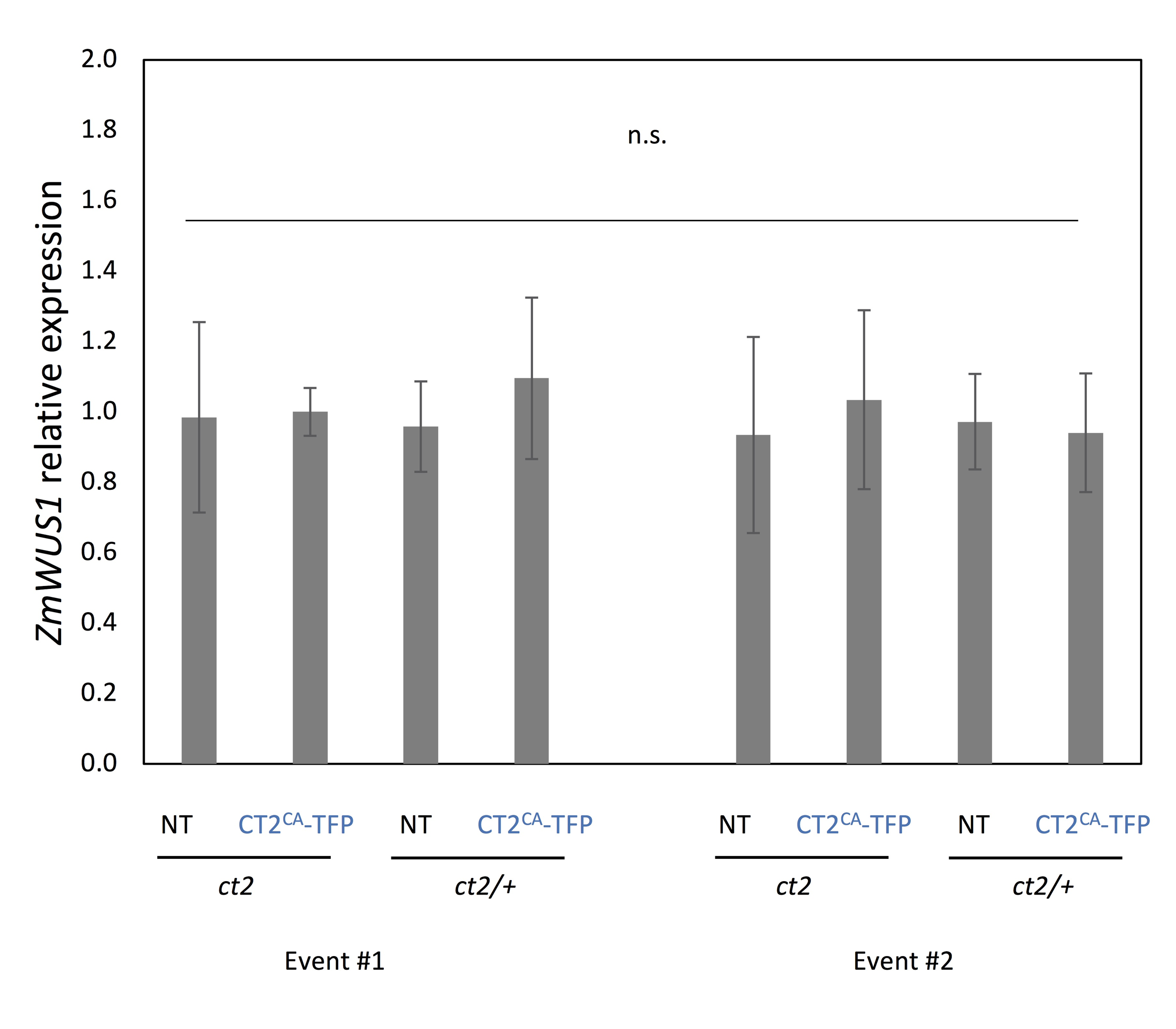

Supplement: S4 Fig — Data are shown as means; error bars represent S.D.; n = 3 biological replicates. The tissues were collected from 4-wk old maize shoot apices. Each replicate contains pooled samples from 4–6 plants. (TIF) [file pgen.1007374.s004.tif]

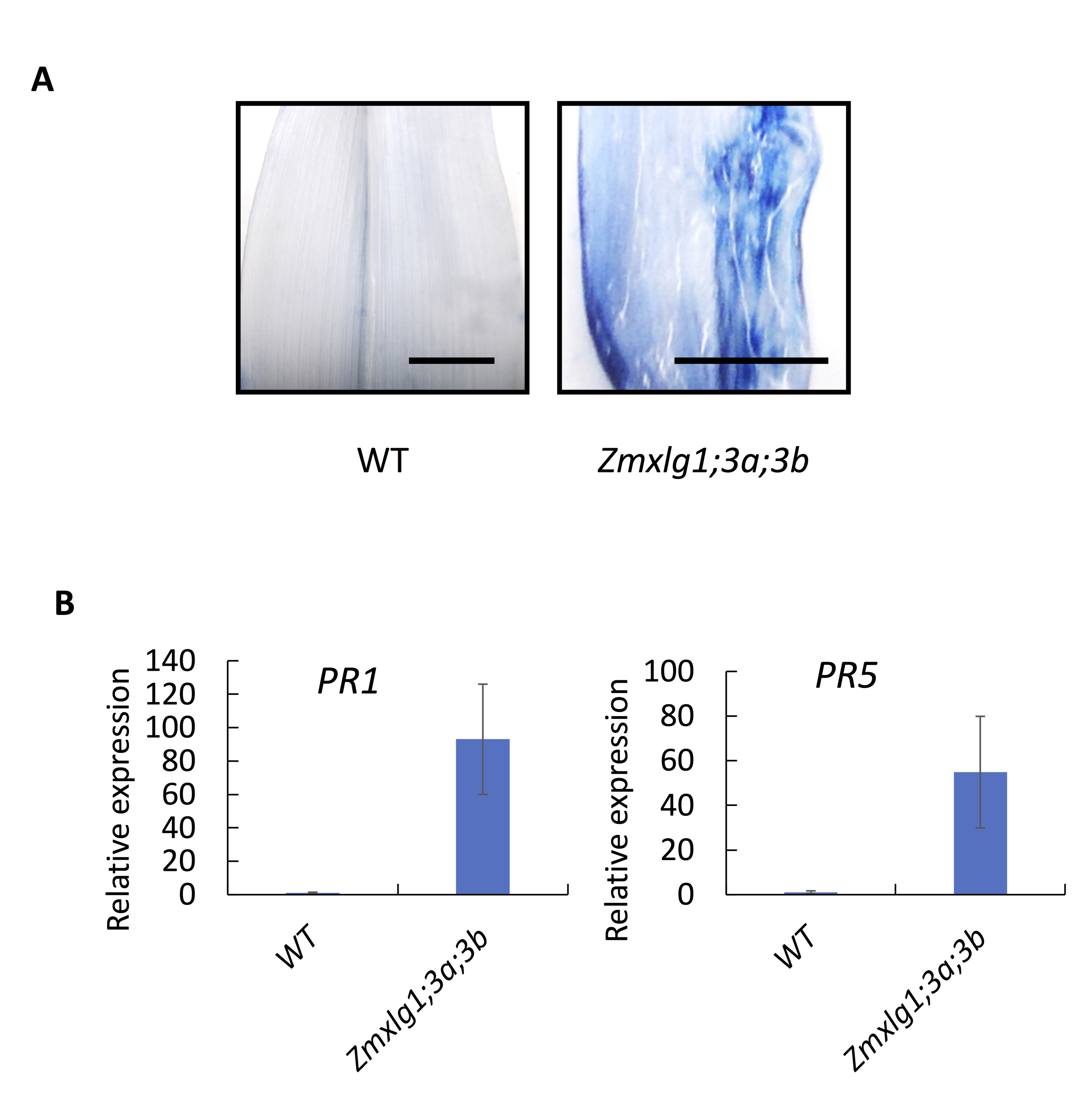

Supplement: S5 Fig — (A) Trypan blue staining of fully expanded wild-type (WT) and Zmxlg1;3a;3b triple mutant leaf blade showed increased staining in the triple mutants, scale bar = 3 mm. Note, the mutant leaf is smaller because of the early growth arrest. (B) PR1 and PR5 expression is massively up-regulated in the Zmxlg triple mutants. Values were normalized to the expression of ZmUBIQUITIN. Error bars represent S.D.; n = 3 biological replicates; p < 0.01 in a Student’s t-test. (TIF) [file pgen.1007374.s005.tif]

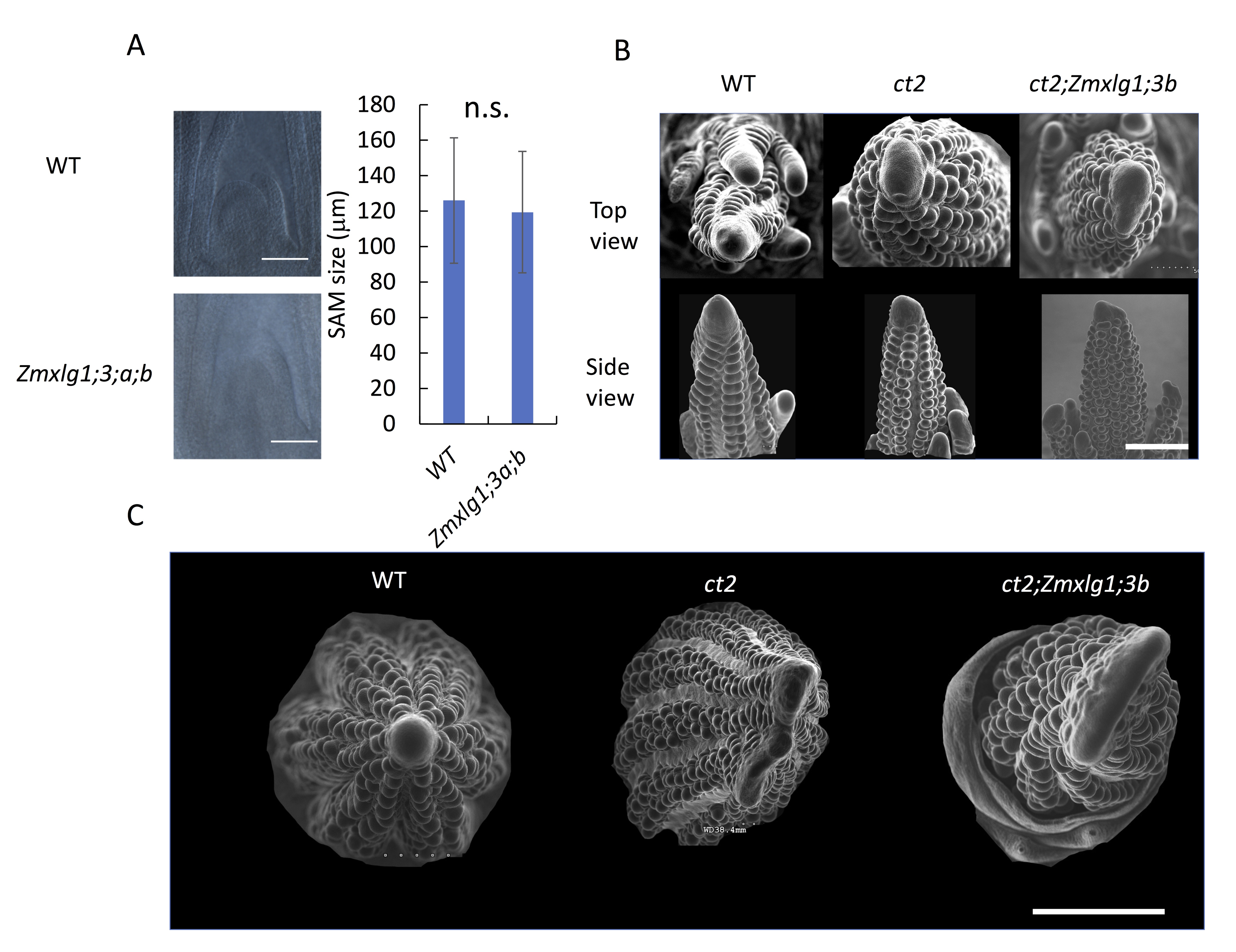

Supplement: S6 Fig — (A) Zmxlg123 triple mutants displayed normal shoot apical meristems. WT, wild-type. Scale bar = 100 μm. Data are shown as means; error bars represent S.D.; n.s. indicates not significantly different (p-value>0.05) in a student’s t-test. Knocking out ZmXLGs in a ct2 mutant background did not enhance the fasciation phenotype of either tassel (B) or ear (C) primordia. Scale bar = 500 μm. (TIF) [file pgen.1007374.s006.tif]

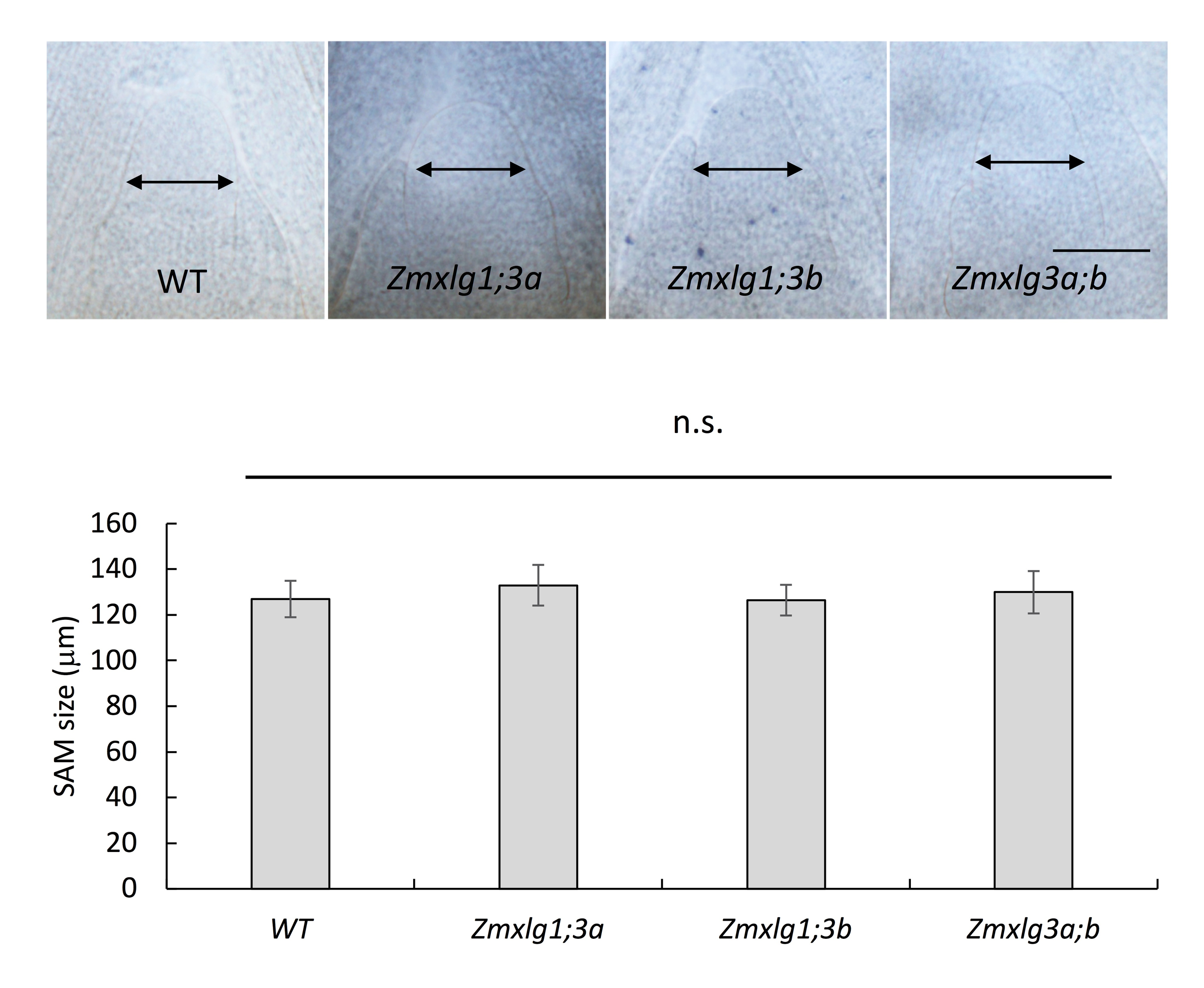

Supplement: S7 Fig — Scale bar = 100 μm. Data were analyzed using ANOVA followed by the Fisher’s LSD test. n.s. indicates not significantly different (p-value>0.05). Data are shown as means; error bars represent S.D.; n = 9–23. (TIF) [file pgen.1007374.s007.tif]

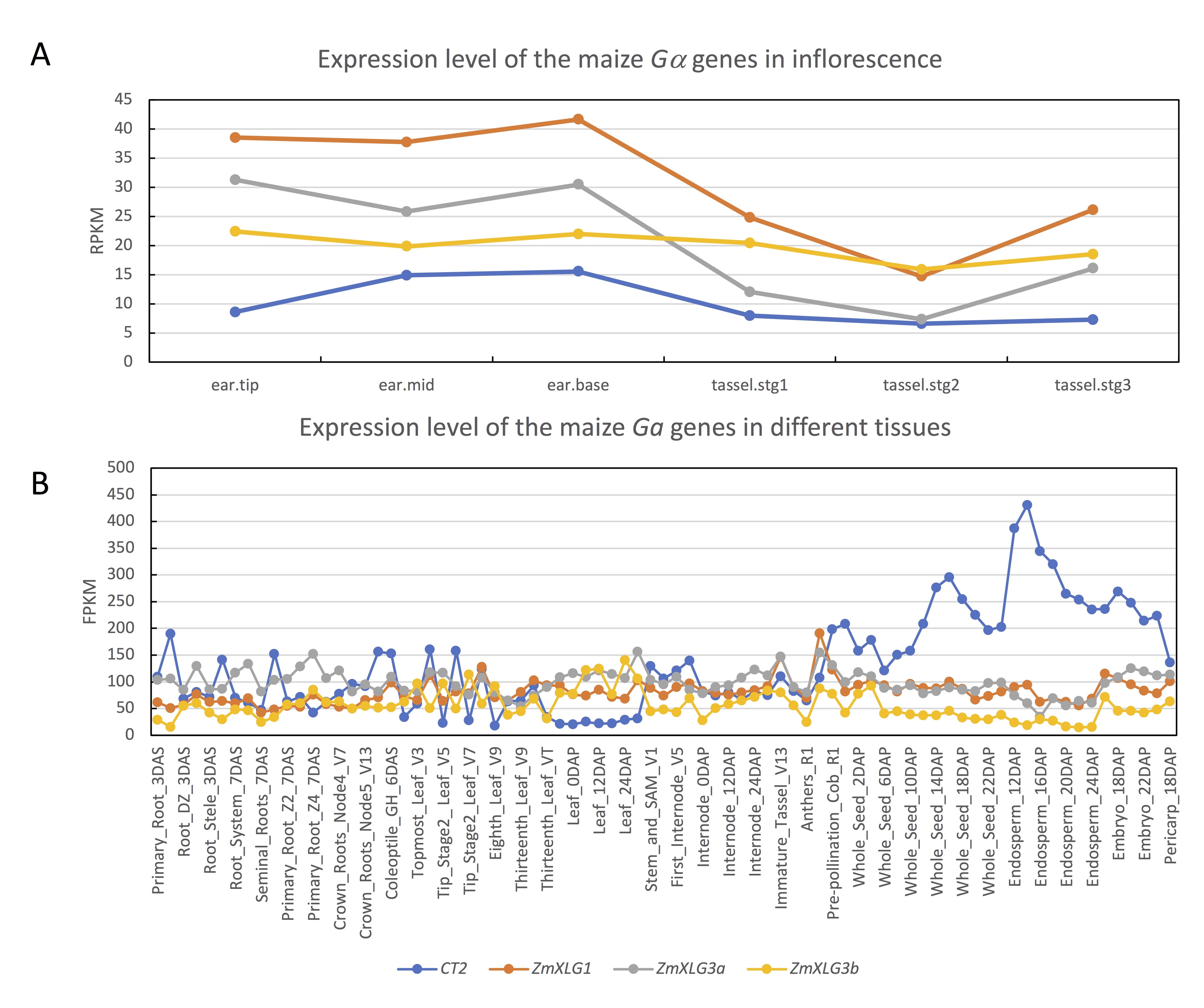

Supplement: S8 Fig — (A) Expression of CT2 and ZmXLGs in the maize inflorescence. The data were mined from www.maizeinflorescence.org and Reference 1 in S1 File Eveland et al., 2014. (B) Expression of CT2 and ZmXLGs in the different tissues at different developmental stages. The data were mined from www.maizegdb.com and Reference 2 in S1 File Stelpflug et al., 2016. (TIF) [file pgen.1007374.s008.tif]
