# Supplementary material for: Role of heterotrimeric Gα proteins in maize development and enhancement of agronomic traits
Source: PLoS Genet. 2018 Apr 30;14(4):e1007374. doi: 10.1371/journal.pgen.1007374 (PMC5945058; doi:10.1371/journal.pgen.1007374)
Supplement: S1 Table — (DOCX) [file pgen.1007374.s009.docx]

**Supplementary Table S1** List of the primer sequences

| **Primer name** | **sequence** | **purpose** |
| --- | --- | --- |
| attB1-gCT2-F | GGGGACAAGTTTGTACAAAAAAGCAGGC TGAGAAAGAGAGACGGTGTTTGGT | Amplify *CT2* genomic region |
| attB4-CT2(324)-R | GGGGACAACTTTGTATAGAAAAGTTGGGTGGGCTAACTCTTTAGCTCCCT | Amplify *CT2* genomic region |
| attB3-CT2(325)-F | GGGGACAACTTTGTATAATAAAGTTGACCAAGTGGAACCAGATTCTTC | Amplify *CT2* genomic region |
| attB2-gct2-R | GGGGACCACTTTGTACAAGAAAGCTGGGTATGACCCGTACCTGAACATCTTCCGGG | Amplify *CT2* genomic region |
| gCT2CA-F | CTGTACGATGTAGGAGGCCTGAGAAACGAGAGAAGGAAATGGATC | *CT2^CA^* PCR-based mutagenesis |
| gCT2CA-R | CATTTCCTTCTCTCGTTTCTCAGGCCTCCTACATCGTACAGCCT | *CT2^CA^* PCR-based mutagenesis |
| mTFP-500-F | CCGACATTAGCATGGAGGAGGAT | Genotype *CT2^CA^-mTFP1* transgene |
| gCT2-behind | GCTGCCTTCTCTGATCAAATGCT | Genotype *CT2^CA^-mTFP1* transgene |
| ct2-GP-F2 | TCATCAAGACAGCAAGTCACGGA | Genotype *ct2* |
| ct2-GP-R2 | TGGGACTTGCAGAATACTCCCAC | Genotype *ct2* |
| EcoRI-CT2-F: | GATCAGAATTCAAATGGGCTCATCCTGTAGCAGA | Amplify *CT2* cDNA for cloning into pPROEX-His |
| XhoI-CT2-R | GTGATCTCGAGTTATCAAGTTCCTTCTCTGGAAC | Amplify *CT2* cDNA for cloning into pPROEX-His |
| CT2qPCR_F2 | AGGAGCTACACATCAGTCATCCA | qPCR for CT2 |
| CT2qPCR_R2 | TGGGTATTCCAATCTGGCACCAA | qPCR for CT2 |
| mTFP1qPCR_R2 | TAACCCCCATTGTGGTCTCCTC | qPCR for CT2CA-mTFP1 |
| YFPqPCR_R2 | GAACTTGTGGCCGTTTACGTCG | qPCR for CT2-YFP |
| ZmWUS1_F | CTCTCGTTGTCGCCTTCTTC | qRT-PCR for ZmWUS1 |
| ZmWUS1_R | ACTCGTGTCCAGCATCACAG | qRT-PCR for ZmWUS1 |
| ZmUBIQUITIN_F | TAAGCTGCCGATGTGCCTGCG | qRT-PCR for Ubiquitin |
| ZmUBIQUITIN_R | CTGAAAGACAGAACATAATGAGCACAG | qRT-PCR for Ubiquitin |
| PR1_qPCR_F | TCAGCAAACAACAAACAATGG | qRT-PCR for PR1 |
| PR1-qPCR_R | GTAGTCCTGCGGCGAGTTCT | qRT-PCR for PR1 |
| PR5_qPCR_F | CGACATGAAGACCCATGCATG | qRT-PCR for PR5 |
| PR5_qPCR_R | CCTGCAAAATCCAAATCACTAGCCCA | qRT-PCR for PR5 |
