# Supplementary material for: Role of heterotrimeric Gα proteins in maize development and enhancement of agronomic traits
Source: PLoS Genet. 2018 Apr 30;14(4):e1007374. doi: 10.1371/journal.pgen.1007374 (PMC5945058; doi:10.1371/journal.pgen.1007374)
Supplement: S1 File — (DOCX) [file pgen.1007374.s010.docx]

**Sequence of the ZmXLG multiple-gRNA array.**

CTTTTGTTTCACTTATCAGTTTTCACCAAGATTGCATGCCACTTGGCGGGTTCATCACGAAGAACCTTCCAATAATGCAAAACTGTGGACGGACCTTTTTTTAGAATAATCGTTCTAACAAAATTGCATAGCCCTGTCTGTCTCTTAGTTGATCATCAGATTGGCCAGTAGCATAAGCTGAGGTAGCCTTGGTCCATGCGGCAAAAAAAAAAGAACCATCTCTCTCTCTTTTGCCAGCCAGGTACACTATTGTGTTTTACCTCTTTTTTAGTCTAAAAAATACTTGTCATTTATTTTCTATCCAGCTGACAATCTTTATGGCCCCTGACGAAATAGTTCGTGGCAAACTGGCAATGGTCTTTTGCAAAACACGACGCTGTGCACGTCAGCACGTGAGCTTGCGATGTCCACTAGGGAGCTCCATCCACTGATCCACCCCCACGCGGCGTGGCGTCGTCATTAACGGCTTGTGGGGAAGGGAACGAGCAACTAACCGATAATTAGTACCAGACCGGCCAGTGAACGATGCCAAAACCGGCTTATAAGCTCAGCTGCGACAACCGTTTTGCGCACGCGGAGGCCATGGAgttttagagctagaaatagcaagttaaaataaggctagtccgttatcaacttgaaaaagtggcaccgagtcggtgcttttttttacgtaCAAAAACATCCTCACAGGAAAGACACGAAGAAACATGGTCAATGGCCCATTATATAAAGCACCGCCACAAAGCCCAAATACCAGTTCGTCGGTGGAGCAAGTAACGCGCTAGGCAACAGGCAAACAGTTTGTCCCACCTCGTCCAGTCACAAAGGCAAAGCGTGACTTATAAGCCAGAGCGGAAGAACCATACCGATGCGGAGGATCCTTCCCCgttttagagctagaaatagcaagttaaaataaggctagtccgttatcaacttgaaaaagtggcaccgagtcggtgctttttttgttaacCACGTGAGCTTGCGATGTCCACTAGGGAGCTCCATCCACTGATCCACCCCCACGCGGCGTGGCGTCGTCATTAACGGCTTGTGGGGAAGGGAACGAGCAACTAACCGATAATTAGTACCAGACCGGCCAGTGAACGATGCCAAAACCGGCTTATAAGCTCAGCTGCGACAACCGTTTTGGAGCGTGTAGGGGAGTGGCgttttagagctagaaatagcaagttaaaataaggctagtccgttatcaacttgaaaaagtggcaccgagtcggtgctttttttgccggcCATGGACAGGACACACTGCTACTACTATAACTGTGTTGTAGACTTGTATAAACAGCAAATAATGGCATCCGATGTGAAGCGACAGTGTATTATATGTATTGTACTGTGTCATAGAGAGCTAATGCCAAAATATCTGAATGGAATGTATGTGTGCCCTGCTTCACCGGGAATCGATAAGAATTTGGGAATTTGGATTCGGTGTTCTTTATTAGGTTCTCGCCGAATATGGTTCTCAGTTATGACCTAACGGTGTCCACAAGAGTTCGCCAGGATTTATACAACTATTTTCTTATTTATTTCTTTAACATTTTCCCTTCTACGCACAATAGGAGATAATGTCAAGCGTTGACGGTGCACATATATTTGTTTTTTTAAAGGCGTAGTGGCGTGTGTGCAAAAACATCCTCACAGGAAAGACACGAAGAAACATGGTCAATGGCCCATTATATAAAGCACCGCCACAAAGCCCAAATACCAGTTCGTCGGTGGAGCAAGTAACGCGCTAGGCAACAGGCAAACAGTTTGTCCCACCTCGTCCAGTCACAAAGGCAAAGCGTGACTTATAAGCCAGAGCGGAAGAACCATACCGGGAGCTCGTACGACACGTCgttttagagctagaaatagcaagttaaaataaggctagtccgttatcaacttgaaaaagtggcaccgagtcggtgcttttttt

Red: Maize U6 promoter 1

Yellow: Maize U6 promoter 2

Cyan: Maize U6 promoter 3

Purple: Maize U6 promoter 4

Green: guideRNAs

Gray: sgRNA

The sequences of the maize U6 promoters were obtained from (Zhu et al., 2016) [[3](#_ENREF_3)].

**Sequences of the yeast-codon optimized ORFs**

*>ZmXLG1*(GRMZM2G127739)

ATGGCTGCAGGTGCTTCTGATTATTCATTTGCTGCAGAATACGTTGGTCCACCATTGCCATATACTTTACCAAGAGCAAT

TCCATTGGATTTGTCTAGAATACCATTGGCTGCATTATCTTCATCTCCACCAGCTTCTCCATCAGCATCATCTTCACCAT

TGCCAGTTGTTAGACCATTGACTCCATCTTCATTATGTAATGCTATTCATGCTCATGCACATCCAGCTCCAAGATCTGCA

GTTCCAGCTCCAGCTGGTGGTGGTGCTGTTGTTGATTCTCCAACATCAGTTATTGAAAATCATCATGCTGCAGCTCATCA

TTCTGCAGAATTACCATCTTCACCATCAGATGATGAAGCAGCTGGTGACGGTGACGGTGACGGTGGTTCTGAAGCTTTGC

CATTAAAACCAAGACATCATCCACCAGTTGTTACTTTTGCAGAAACATCTGGTTCATTGTTGCAATCTTCAGATGATGAA

GATGAATACGAAGAAGAAGATGATGATGATGATGATGCTGATGCAGGTGAAACTAGACCAAGAGCAGCTGCAGGTCAATC

TTCAGGTTCTTTGTCACCAGCTCATTGGAGAGGTGGTAGAACAAGAGGTTGTTATAGATGTGGTAAAGGTGGTGGTTTTT

GGGGTAGAGATAAAGAATCTTGTTTGGCTTGTGGTGCAAGATACTGTGTTGGTTGTGTTTTAAGAGCTATGGGTTCTATG

CCAGAAGGTAGAAAATGTTTGGAATGTATTGGTAGACCAGTTGCTGAATCAAGAAGAGATGCATTGGGTAGAGGTTCTAG

AGTTTTAAGAAGATTGTTATCAGCTGCAGAAGTTGAATTAGTTATGAGATCTGAAAGAGAATGTGCTGCAAATCAATTGA

GAGCTGAAGATGTTTACGTTAACGGTTCTAAGTTGTCACCAGAAGAATTGGTTGTTTTACAAGGTTGTCCATGTCCACCA

TCTAGATTAAGACCAGGTTTCTACTGGTACGATAAAGTTTCAGGTTTTTGGGGTAAAGAAGGTCATAAGCCACATTGTAT

CATCACTGCTAATTTGAATGTTGGTGGTTCTTTGGATCAAAAGGCATCAAACGGTAACACTGGTATTTTGATTAATGGTA

GAGAAATCACAAAATCTGAATTACAAATGTTGAAATTAGCTGGTGTTCAATGTGCAGGTAAACCACATTTTTGGGTTAAT

GCTGATGGTACTTACCAAGAAGAAGGTCAAAAGACTGTTAAGGGTAAAATCTGGGATAAGCCAATCGTTAAGTTGTTGTC

TCCAGTTTTGTCATTACCAACACCAAATAAGGCTGCAAATCAATCTTCAGAAGATGCTGTTGATATCGTTAACAGAGCAA

TCCCAGATTATTTGGAACAAAGAACTACACAAAAGTTTTTGTTAGTTGGTTCTGGTGCTTCAACTATCTTGAAGCAAGCA

AAGTTCTTGTACAAAACAGGTAAACCATTTTCTGTTGATGAAAGAGAAGATTTGAAGTTGATCATCCAATCAAACATCTA

TAACTACTTGGGTATTTTGTTGGAAGGTAGAGAAAGATTCGAAGAAGAATCTTTGGCTGATAGAAGAAAGACTTCACAAT

GTGATCCATCTTCATCTGGTTGTTGTGAACCAGGTTTGTGTGATGAAGTTACAGAATACTCTTTGATCCCAAGATTGAAG

GCTTTTTCAGATTGGATCTTGAAAGCTATGGCATTGGGTAATTTGGAAGATATTTTTCCAGCTGCATCTAGAGAATACGC

ACCATTGGTTGATGAATTATGGAAAGATCCAGCTATCCAAGCAACTTACAGAAGATCTGAATTGCCATTTTTACCACCAG

CTGCATCATACTTTTTGGATAAGGCTGTTGATATCTCTAGAACAGAATACGAATTGTCAGATATGGATATCTTGTACGCT

GATGGTATCACTTCATCTGATGGTGTTGCATCTACAGAATTTTCATTCCCACAATTGTCTTTAGGTGGTTTGGGTGTTGA

TGAACCAGATGCTCAAGATACTTTGTTGAGATACCAATTGATCAGAATTAATTCAAGAGGTTTGCATGGTAACTGTAAGT

GGTTGCAAATGTTCGATGATGTTAGATTGGTTATTTTCTGTGTTGCTGCATCTGATTACGATGAATATTACGAAGATGCT

AACGGTACTATCGTTAATAAGATGATCGAATCTAGACAATTGTTCGAATCAATCACTTTACATCCAACATTCGAACAAGC

AGATTTCTTGTTGTTGTTGACAAAGTTCGATTTGTTAGAACAAAAGATTTCATCTTCACCATTGACTTCTTGTGATTGGT

TTTCAGATTTCACACCATTAATCTCTAGAAATTTGTTAAACGGTGGTGGTGGTGGTGGTAGAAGAACAGCTAGATCTTCA

ACTGGTGCAACATTGCCACAAATGGCTGCACATTATATGGCTACTAAGTTTAAAAGATTGTTCGAATCTATGACAGGTAG

AAAGTTGTACGTTTCATACGTTAACGCTTTGGATCAAGAATCTGTTAGATCAGCAATCAGATACGGTAGAGAAGTTGTTA

AGTGGGAAGAAGAAAAACCAGTTTTTGGTGCTTCTGAAACTGTTTACTCAGAAGAACCATCTTCTTTTACTGCATAA

*>ZmXLG3a* (GRMZM2G016858)

ATGGCTGAAGCACATGCTGAAGCAATGGATGGTTCAACTTGGGAAGATATGATGAGAAGAATTTTGCCACCAGGTACACC

AATTCCAGAAGCTCCACCAAATTTGGATTACTCTATCGCATTGGAATATGATGGTCCACCAGTTCCATACGAATTACCAA

GAGTTGATCCAGTTGAAATTCCAGCTATTCCAACTGCAGAACCAGTTTCAGGTTCTCAAGTTTTGGGTGGTTTACCAGTT

GCTCCAGTTGTTCAACCAATTAGATTGCCAGTTTCTAGAATTGCTAGATGTGCAGATCCAGTTGCTGCACAAGCTGCAGG

TTCTTCAGAATCAGTTTTGCATAACCAAGAATTTTCTGATGATGATGAAGAAGGTGACGATTCAAGATCTCAATCACATG

GTTCAGCTCAATCTTCACCAGGTCCACAAAATAGACCAGAAAGACAAGAAGGTAGAAGAGGTCCAGTTGTTACTTTCGGT

TTTACACCAGATTCTAAGTACGAATCAAAGGAATTTGAAGAAATGTCTGAACAATACGTTGCTGTTACTAAGAAAGAAAA

GAGAAGAAGAGCATGTTATAGATGTGGTAAAAGAAAGTGGGAATCTAAGGAATCATGTTTGGTTTGTGATGCTAGATACT

GTGGTTACTGTGTTTTGAGAATGATGGGTTCTATGCCAGAAGGTAGAAAGTGTGTTAACTGTATCGGTCAACCAATCGAT

GAATCTAAGAGATCAAAGTTGGGTAAAAATTCTAGAACTTTGTCAAGATTGTTGTCTCCATTGGAAGTTAGACAAATCTT

GAAGGCTGAAAAGGAATGTCAAGCAAACCAATTGAGACCAGAACAATTGATCGTTAACGGTTGTCCATTGAGACCAGAAG

AATTGACAGATTTGTTGTCTTGTTCAAGACCACCACAAAAATTGAAACCTGGTAAATACTGGTACGATAAGGAATCAGGT

TTATGGGGTAAAGAAGGTGAAAAGCCAGATAGAATCATCTCTTCAAATTTGAACTTCACTGGTAAATTGCAAGTTAACGC

TTCTAACGGTAACACTCAAGTTTACATGAATGGTAGAGAAATCACAAAGATCGAATTGAAGGTTTTGAAGGTTGCAAACG

TTCAATGTCCAAGAGATACACATTTTTGGGTTTACGATGATGGTAGATATGAAGAAGAAGGTCAAAACAACATCAAGGGT

AAAATCTGGGAATCTGCTTTGGCAAGATTTGCTTGTGCATTGTTTTCATTACCAGTTCCACCAGGTAATTCTAATGGTAC

TAGAGATGAAGTTCCATACACAGCTAGAGCAGTTCCAGATTATTTGGATCAAAAGAGAATACAAAAGTTGTTGTTGTTGG

GTCCACCAGGTGCTGGTACTTCAACAATTTTTAAGCAAGCAAAGTACTTGTATGGTACTAGATTCACTCAAGAAGAATTG

GAAGGTATTAAATTGATGATCCAATCTAACATGTTTAAATACTTAGGTATTTTGTTGGAAGGTAGAGAAAGATTCGAAGA

AGAAGCTTTGTCAAGATTGAACTACACTATCTCTCAAGGTGAAGAAACACAACATGATGAAAATAAGGCTAACGGTTCTA

ATTCATGTATCTATTCAATTAATGCAAGATTGAAGAAATTTTCTGATTGGTTGTTGGATATCATCGCTATGGGTGACTTG

GATGCTTTCTTTCCAGCTGCAACTAGAGAATATGCTCCATTTGTTGATGAAATGTGGAAAGATCCAGCTATTCAAGCAAC

TTTTAAAAGAAAGGAAGAATTGCATTTCTTGCCAGATGTTGCTGAACATTTCTTGTCAAGAGCAGTTGAAGTTTCTTCAA

ACGAATACGAACCATCTGAAAAGGATGTTATCTTCGCTGAAGGTGTTACTCAGGGTAACGGTTTGGCTTTTATTGAGTTT

ACTTTGGATGATAGATCTCCAATGTCAGAACCATACATTGATAATTCAGAAGCTCATTCTCAACCATTGACTAAGTACCA

ATTGATCAGAGTTTCTGCAAAGGGTATGAATGATGGTTGTAAGTGGGTTGAAATGTTCGAAGATGTTAGAATGGTTATTT

TCTGTGTTGCTTTGTCAGATTACGATCAATTAGGTCCACCAGTTTCTGGTTCTTCAAGACCATTGGTTAATAAGATGATG

CAATCTAAGGAATTGTTCGAAGCTACTATCAGACAACCATGTTTTTGTGATACACCATTCGTTTTGGTTTTGAATAAGTA

CGATTTGTTCGAAGAAAAGATTAATAGAGCACCATTATCTTCATGTGAATGGTTCAACGATTTCTGTCCAGTTAGAACAC

ATCATAACAACCAATCATTGGCTCATCAAGCATACTACTACGTTGCTATGAAGTTTAAAGATTTGTACTCTGCACAAACT

AACAGAAAGTTGTTCGTTTGGCAAGCTAGAGCAAGAGAAAGACAAACAGTTGATGAAGCTTTTAAATACATCAGAGAAGT

TTTGAAGTGGGAAGAAGAAAAGGATGATAACTGTTACCAAGAAGAATCTTTCTACTCAACTACAGAAATGTCTTCATCTC

CTTTTATTAGAGCAGAATAA

>ZmXLG3b (GRMZM2G429113)

ATGGCTGCAGGTAGAGGTGACTGGGAAGATATGGTTAGAAGAATGTTTCCACCAGGTACTACAATTCCAGAACCATTGCC

AGATATGGATTACTCATTCGCATTGACTTACAACGGTCCAGATGTTTCTTATGAATTGCCAAGAATACAACCAGTTTGTG

TTCCAGCTATTCCAACAGCTGAACCAGCATCAGGTCCATTGGGTTTAGGTTCTGGTGTTGTTCCAGTTGCACCAGTTGTT

GGTCCAGCTGCAGCTAGAGCTAGAGCAAATCCACCAGCAGCTTCAAGAGCTGGTGCAGCTGATAGAAGAGCAGCTAGAAT

GACTCCATCTGATTCTTCAGATGATGAAGAAGATGATGCTGGTTCTTCAGGTTCAAGATCTACTAAAGCTTCTAGACCAG

CAGTTCCAGAAGGTAGAAGACCACAAGTTGTTACATTTGGTGTTCCAGAAGAAGATTCAGGTGGTAGATATGAATCTTCA

GAAATGGATGGTGGTCATGATGCAGCTTCTGCTGAACAACATGTTGCAGTTACTAGACCAGCTGAAAGAAAAGGTAGAAC

ATGTTGTAGAAGATGTGGTAAATCTAAGTGGGAATCAAAGGAATCTTGTATCGTTTGTGATGCAAGATACTGTGGTCATT

GTTTGTTAAGAGCTATGGGTTCAATGCCAGAAGGTAGAAAATGTGTTACATGTATTGGTCAACCAATTGATGAAGCAAAA

AGATCTAGATTGGGTAAAGGTTCAAGAATTTTATCTAGATTGTTGGGTCCATTGGAAGTTAGACAAATCTTGAAGGTTGA

AAAGGAATGTCAAGCTAACCAATTGAGACCAGAACAATTGTTGGTTAACGGTTTCCCATTGGATGATGAAGAAATGACTG

ATTTGTTGTCATGTCAAAGACCACCAGGCAATTTGAAACCAGGTAGATATTGGTACGATAAAGAATCTGGTTTATGGGGT

AAAGAAGGTGAAAAGCCAGATTCAATCATCTCTACAAATTTGAACTTCAACGGTAAATTGCAACCAGATGCATCTAACGG

TACTGCTCAAGTTTTTATTAATGGTAGAGAAATCACAAAGATCGAATTGAGAATTTTAAAAATTGCTAAAGTTCAATGTC

CAAGAGATACTCATTTTTGGGTTTATCATGATGGTGGTTACGAAGAAGAAGGTCAAAACAACATCAAGGGTAAAATCTGG

GAATCACCATTGACAAGATTTGCTTGTGCATTGGTTTCTTTACCAGTTCCACCAACTAATTTTGATGCAACAAAAGATGA

AGCTCCATACTCTTCAAAGACTGTTCCAGATTACTTGGATCATAAGAGAATACAAAAGTTGTTGATCTTAGGTTCACCAG

GTGCAGGTACTTCTACAATTTTTAAGCAGGCTAAGTTGTTGTACGGTAACAGATTCACTGATGAAGAATTGGAAAACATC

AAGTTGATGATCCAATCTAACATGTTCAAGTACTTGGGTATTTTGTTGGAAGGTAGAGAAAGATTCGAAGAAGAAGCTTT

AGCAGTTCCAAATCATCCATCTTCAGTTGGTGACGATCCACAACAAGATGAATCAAAATCTTCATCTTCAAATTCATGTA

TCTATTCTATTAATGCAAAGTTGAAGAAATTTTCTGATTGGTTGTTGGATATCATCGCTACTGGTGACTTGGATACTTTC

TTTCCAGCAGCTACTAGAGAATACGCTCCAGTTGTTGATGAATTGTGGAAAGATCCAGCTATTCAAGCAACATACAAGAG

AAAGGATGAATTGCATTTCTTGCCAGATGCAGCTGAATACTTTTTATCAAGAGCTGTTGAAGTTTCTTCAAACGAATACG

AACCATCTGAAAAGGATGTTATCTATGCAGAAGGTGTTACTCAGGGTAACGGTTTGGCTTTTATTGATTTCACATTGGAT

GATAGATCTCCAATGTCAGAATCTTTTGGTGACAATCATGATGCATATCCACAACAACCAGTTAATAAGTACCAATTGAT

CAGAGTTTCAGCTAAGGGTATGAATGAAGGTTGTAAATGGGTTGAAATGTTCGAAGATGTTTCTATGGTTATTTTCTCTG

TTGCTTTGTCTGATTATGATCAATTAGGTGCTCCAGCATCAGGTTCTTCAAGAACTTTGGTTAATAAGATGATCCAATCT

AGAGATTTGTTCGAAGCAACTATCAGACAACCATGTTTTAGAGATACACCATTTTTGTTGTTGTTGAATAAGTACGATGC

TTTTGAAGAAAAGATTGGTAGATCTCCATTGTCTTCATGTGAATGGTTTGGTGACTTTTGTCCATTGAGAACTCATCATA

ACTCACAATCTTTGGCTCAACAAGCATTCTACTACGTTGCTATTAAGTTTAAAGATTTGTACGCAGCTTCTACAGGTAGA

AAATTATTTGTTTGGCAAACTAGAGCAAGAGATAGACCAACAGTTGATGAAGCTTTTAGATACGTTAGAGAAGTTTTGAG

ATGGGAAGATGAAAGAGGTGTTGCTGGTTATTGTCCAGATGAATCATTCTACTCTACTACAGAATTGTCTTCATCTAGAT

TGATTGCAGCTGCACAATAA

>*ZmRGG2* (GRMZM6G935329)

ATGCAAGTTGGTGACGGTGGTGGTGACTCTGCTGATTTGAGAGGTAGACATAGAATCCAAGCTGAATTGAAGAAATTGGA

ACAAGAAGCAAGATTTTTAGAAGAAGAATTGGAAGAATTGGATAAGGCTGATAAAGTTTCTTCAGCATTGCAAGAATTCT

TGATCGCTATGGAAAGAAAGGCAGATCCATTGTTACCAGTTTCTGCAGGTCCAGTTAATCAATCATGGGATAGATGGTTT

GAAGGTCCACAAGATTTGAGAGGTTGTAAATGTTGGTTTTTATAA

>*ZmGB1* (GRMZM2G045314)

ATGGCTTCAGTTGCAGAATTGAAAGAAAAACATGCTGCAGCTACTGCTTCTGTTAATTCATTGAGAGAAAGATTGAGACA

AAGAAGAGAAACTTTGTTGGATACAGATGTTGCAAGATACTCTAAGTCACAAGGTAGAGTTCCAGTTTCTTTTAATCCAA

CTGATTTGGTTTGTTGTAGAACATTGCAAGGTCATTCTGGTAAAGTTTACTCATTGGATTGGACACCAGAAAAGAATTGG

ATTGTTTCTGCTTCACAAGATGGTAGATTAATTGTTTGGAATGCTTTGACTTCTCAAAAGACTCATGCAATTAAATTGCA

TTGTCCATGGGTTATGGCATGTGCTTTTGCACCAAATGGTCAATCTGTTGCTTGTGGTGGTTTGGATTCTGCATGTTCAA

TTTTTAATTTGAACTCTCAAGCAGATAGAGATGGTAATATGCCAGTTTCAAGAATTTTGACTGGTCATAAGGGTTACGTT

TCTTCATGTCAATACGTTCCAGATCAAGAAACTAGATTGATTACATCTTCAGGTGACCAAACATGTGTTTTATGGGATGT

TACTACAGGTCAAAGAATCTCTATCTTCGGTGGTGAATTTCCATCAGGTCATACTGCTGATGTTCAATCTGTTTCAATTA

ATTCTTCAAACACAAACATGTTTGTTTCTGGTTCATGTGATACTACAGTTAGATTGTGGGATATCAGAATCGCTTCTAGA

GCAGTTAGAACTTATCATGGTCATGAAGATGATGTTAATTCTGTTAAGTTTTTCCCAGATGGTCATAGATTTGGTACTGG

TTCAGATGATGGTACATGTAGATTGTTCGATATGAGAACAGGTCATCAATTGCAAGTTTACTCTAGAGAACCAGATAGAA

ATTCAAACGAATTGCCAACTGTTACATCTATCGCTTTTTCTATCTCAGGTAGATTGTTATTTGCAGGTTATTCAAATGGT

GACTGTTACGTTTGGGATACTTTGTTGGCTGAAGTTGTTTTGAATTTGGGTAATTTGCAAAATTCTCATGATGGTAGAAT

CTCATGTTTAGGCATGTCTTCAGATGGTTCTGCTTTGTGTACAGGTTCATGGGATAAGAATTTGAAAATTTGGGCATTTT

CTGGTCATAGAAAAATTGTTTAA

>*CT2* (GRMZM2G064732)

ATGGGTTCTTCATGTTCTAGATCACATTCTTTTGATGAAGCTGAAGCTGCAGAAAACGCTAAGTCTGCAGATATCGATAG

AAGAATTTTACAAGAAACTAAGGCTGAACAACATATCCATAAGTTGTTGTTGTTAGGTGCTGGTGAATCTGGTAAATCTA

CTATTTTTAAGCAAATTAAATTGTTATTTCAAACAGGTTTTGATGAAGCTGAATTGAGATCATACACTTCTGTTATTCAT

GCAAACGTTTACCAAACAATTAAAATCTTGTACGAAGGTGCTAAGGAATTAGCACAAGTTGAACCAGATTCTTCAAAGTA

CGTTTTGTCACCAGATAACCAAGAAATCGGTGAAAAGTTGTCAGAAATCGGTGCAAGATTGGAATACCCATCTTTGAATA

AGGAAAGAGTTCAAGATGTTAGAAAATTGTGGCAAGATCCAGCTATTCAAGAAACTTACTCAAGAGGTTCTATTTTACAA

GTTCCAGATTGTGCACAATACTTCATGGAAAATTTGGATAAGTTGTCTGAAGAAGATTACGTTCCAACAAAAGAAGATGT

TTTACATGCTAGAGTTAGAACTAATGGTGTTGTTGAAACACAATTTTCACCATTGGGTGAATCTAAGAGAGGTGGTGAAG

TTTACAGATTGTACGATGTTGGTGGTCAAAGAAACGAAAGAAGAAAGTGGATCCATTTGTTCGAAGGTGTTAACGCTGTT

ATTTTCTGTGCTGCAATTTCTGAATATGATCAAATGTTGTTCGAAGATGAAACTAAAAATAGAATGATGGAAACAAAAGA

ATTGTTTGATTGGGTTTTAAAACAAAGATGTTTTGAAAAGACTTCTTTTATGTTGTTTTTAAATAAGTTCGATATTTTTG

AAAGAAAAATTCAAAAAGTTCCATTGTCTGTTTGTGAATGGTTCAAGGATTACCAACCAACAGCTCCAGGTAAACAAGAA

GTTGAACATGCATACGAATTTGTTAAAAAGAAATTCGAAGAATTGTACTTTCAATCTTCAAAGCCAGATAGAGTTGATAG

AGTTTTTAAAATCTATAGAACTACAGCTTTGGATCAAAAGTTGGTTAAGAAAACTTTTAAATTGATTGATGAATCAATGA

GAAGATCTAGAGAAGGTACATAA

*>CT2^CA^*

ATGGGTTCTTCATGTTCTAGATCACATTCTTTTGATGAAGCTGAAGCTGCAGAAAACGCTAAGTCTGCAGATATCGATAG

AAGAATTTTACAAGAAACTAAGGCTGAACAACATATCCATAAGTTGTTGTTGTTAGGTGCTGGTGAATCTGGTAAATCTA

CTATTTTTAAGCAAATTAAATTGTTATTTCAAACAGGTTTTGATGAAGCTGAATTGAGATCATACACTTCTGTTATTCAT

GCAAACGTTTACCAAACAATTAAAATCTTGTACGAAGGTGCTAAGGAATTAGCACAAGTTGAACCAGATTCTTCAAAGTA

CGTTTTGTCACCAGATAACCAAGAAATCGGTGAAAAGTTGTCAGAAATCGGTGCAAGATTGGAATACCCATCTTTGAATA

AGGAAAGAGTTCAAGATGTTAGAAAATTGTGGCAAGATCCAGCTATTCAAGAAACTTACTCAAGAGGTTCTATTTTACAA

GTTCCAGATTGTGCACAATACTTCATGGAAAATTTGGATAAGTTGTCTGAAGAAGATTACGTTCCAACAAAAGAAGATGT

TTTGCATGCTAGAGTTAGAACTAATGGTGTTGTTGAAACACAATTTTCACCATTAGGCGAATCTAAAAGAGGTGGTGAAG

TTTATAGATTGTACGATGTTGGTGGTTTGAGAAACGAAAGAAGAAAGTGGATCCATTTGTTCGAAGGTGTTAACGCTGTT

ATTTTCTGTGCTGCAATTTCTGAATATGATCAAATGTTGTTCGAAGATGAAACTAAAAATAGAATGATGGAAACAAAAGA

ATTGTTTGATTGGGTTTTAAAACAAAGATGTTTTGAAAAGACTTCTTTTATGTTGTTTTTAAATAAGTTCGATATTTTTG

AAAGAAAAATTCAAAAAGTTCCATTGTCTGTTTGTGAATGGTTCAAGGATTACCAACCAACAGCTCCAGGTAAACAAGAA

GTTGAACATGCATACGAATTTGTTAAAAAGAAATTCGAAGAATTGTACTTTCAATCTTCAAAGCCAGATAGAGTTGATAG

AGTTTTTAAAATCTATAGAACTACAGCTTTGGATCAAAAGTTGGTTAAGAAAACTTTTAAATTGATTGATGAATCAATGA

GAAGATCTAGAGAAGGTACATAA

**References**

1. Eveland AL, Goldshmidt A, Pautler M, Morohashi K, Liseron-Monfils C, Lewis MW, et al. Regulatory modules controlling maize inflorescence architecture. Genome research. 2014;24(3):431-43. doi: 10.1101/gr.166397.113.

2. Stelpflug SC, Sekhon RS, Vaillancourt B, Hirsch CN, Buell CR, de Leon N, et al. An Expanded Maize Gene Expression Atlas based on RNA Sequencing and its Use to Explore Root Development. Plant Genome-Us. 2016;9(1). doi: 10.3835/plantgenome2015.04.0025.

3. Zhu JJ, Song N, Sun SL, Yang WL, Zhao HM, Song WB, et al. Efficiency and Inheritance of Targeted Mutagenesis in Maize Using CRISPR-Cas9. J Genet Genomics. 2016;43(1):25-36. doi: 10.1016/j.jgg.2015.10.006.
